# Loss of lysosomal acid lipase results in mitochondrial dysfunction and fiber switch in skeletal muscles of mice

**DOI:** 10.1016/j.molmet.2023.101869

**Published:** 2023-12-30

**Authors:** Alena Akhmetshina, Valentina Bianco, Ivan Bradić, Melanie Korbelius, Anita Pirchheim, Katharina B. Kuentzel, Thomas O. Eichmann, Helga Hinteregger, Dagmar Kolb, Hansjoerg Habisch, Laura Liesinger, Tobias Madl, Wolfgang Sattler, Branislav Radović, Simon Sedej, Ruth Birner-Gruenberger, Nemanja Vujić, Dagmar Kratky

**Affiliations:** 1 Gottfried Schatz Research Center, Molecular Biology and Biochemistry, Medical University of Graz, Graz, Austria; 2 Department of Biomedical Science, University of Copenhagen, Copenhagen, Denmark; 3 Institute of Molecular Biosciences, University of Graz, Graz, Austria; 4 Core Facility Mass Spectrometry, Center for Medical Research, Medical University of Graz, Graz, Austria; 5 BioTechMed-Graz, Graz, Austria; 6 Core Facility Ultrastructural Analysis, Medical University of Graz, Graz, Austria; 7 Gottfried Schatz Research Center, Cell Biology, Histology and Embryology, Medical University of Graz, Graz, Austria; 8 Institute of Chemical Technologies and Analytics, TU Wien, Vienna, Austria; 9 Department of Internal Medicine, Division of Cardiology, Medical University of Graz, Graz, Austria; 10 Institute of Physiology, Faculty of Medicine, University of Maribor, Slovenia; 11 Diagnostic and Research Institute of Pathology, Medical University of Graz, Graz, Austria

**Keywords:** LAL, LAL deficiency, Lal-deficient mouse, Energy metabolism, Muscle proteomics

## Abstract

**Objective:**

Lysosomal acid lipase (LAL) is the only enzyme known to hydrolyze cholesteryl esters (CE) and triacylglycerols in lysosomes at an acidic pH. Despite the importance of lysosomal hydrolysis in skeletal muscle (SM), research in this area is limited. We hypothesized that LAL may play an important role in SM development, function, and metabolism as a result of lipid and/or carbohydrate metabolism disruptions.

**Results:**

Mice with systemic LAL deficiency (Lal−/−) had markedly lower SM mass, cross-sectional area, and Feret diameter despite unchanged proteolysis or protein synthesis markers in all SM examined. In addition, Lal−/− SM showed increased total cholesterol and CE concentrations, especially during fasting and maturation. Regardless of increased glucose uptake, expression of the slow oxidative fiber marker MYH7 was markedly increased in Lal−/−SM, indicating a fiber switch from glycolytic, fast-twitch fibers to oxidative, slow-twitch fibers. Proteomic analysis of the oxidative and glycolytic parts of the SM confirmed the transition between fast- and slow-twitch fibers, consistent with the decreased Lal−/− muscle size due to the “fiber paradox”. Decreased oxidative capacity and ATP concentration were associated with reduced mitochondrial function of Lal−/− SM, particularly affecting oxidative phosphorylation, despite unchanged structure and number of mitochondria. Impairment in muscle function was reflected by increased exhaustion in the treadmill peak effort test in *vivo*.

**Conclusion:**

We conclude that whole-body loss of LAL is associated with a profound remodeling of the muscular phenotype, manifested by fiber type switch and a decline in muscle mass, most likely due to dysfunctional mitochondria and impaired energy metabolism, at least in mice.

## 1 Introduction

Lysosomal acid lipase (LAL) hydrolyzes cholesteryl esters (CE) and triacylglycerols (TG) at acidic pH [1,2]. Congenital mutations of the LAL-encoding *LIPA* gene cause a substantial reduction in enzyme activity, resulting in a massive accumulation of neutral lipids in lysosomes [3,4], ultimately leading to cellular dysfunction and damage of various cells and organs. The main organs affected by LAL deficiency (LAL-D) are the liver, spleen, adrenal glands, small intestine, and the vasculature [5].

The infantile form of LAL-D, formerly known as Wolman disease, is a severe and rapidly progressive condition that develops within the first few weeks of life. In these patients, LAL activity is 1–2% of mean normal levels, and patients die at a median age of 3.7 months [6]. The disease manifests with nausea, vomiting, intestinal malabsorption, impaired growth and development, and severe liver damage [7], rapidly progressing to fibrosis and cirrhosis [8]. In contrast, late-onset LAL-D, formerly known as CE storage disease, occurs between 2 and 60 years of age and may be latent and asymptomatic; thus, it is often not diagnosed until a routine physical examination and biochemical blood tests are performed. Symptoms include liver damage such as hepatomegaly, elevated alanine and aspartate aminotransferase, steatosis, liver fibrosis, and cirrhosis. In addition, splenomegaly and concomitant pathologies such as anemia and thrombocytopenia may be observed, as well as early development of atherosclerosis [5,9].

In contrast to humans with complete loss of LAL, Lal-deficient (Lal−/−) mice [10] are viable with a median lifespan of almost one year. Similar to LAL-D patients, they exhibit ectopic CE and TG accumulation, particularly affecting the liver, spleen, adrenal glands, and small intestine [11]. Moreover, the animals suffer from growth retardation and progressive loss of white and brown adipose tissue [10]. Lal−/− mice represent a genetic model for early-onset LAL-D but phenotypically and histopathologically more closely resemble patients suffering from late-onset LAL-D [12,13].

Reduced intestinal lipid absorption [14,15], decreased plasma leptin concentrations, and lipodystrophy lead to impaired lipid metabolism in Lal−/− mice, resulting in reduced circulating TG levels in the fasted state [16]. In addition, TG and CE cannot be hydrolyzed in the absence of LAL and remain entrapped in the lysosomes of the Lal−/− liver. To adapt to the resulting reduced availability of fatty acids (FA), glucose consumption is increased, leading to a decrease in plasma and liver glucose levels as well as hepatic glycogen content [16].

The SM is a remarkably malleable organ that undergoes substantial remodeling in response to a wide range of different stimuli such as nutrient content or physical activity [17–19]. Recent studies have suggested that lysosomes play a critical role in maintaining SM mass, particularly as an intracellular signaling hub for activation of the mechanistic target of rapamycin complex 1 (mTORC1) and transcription factor EB (TFEB)/signaling in conjunction with lysosome biogenesis in regulating mTORC1-mediated protein synthesis [20]. However, how impaired lipid processing in lysosomes affects SM has not yet been well described. As SM is not able to store excess lipids like adipose tissue, FA that enter the SM are either oxidized for energy production or used for other metabolic processes. For this reason, lipid degradation by autophagy may be difficult to detect under these conditions [21]. However, FA exported from the lysosome are known to be intensively utilized by the SM for mitochondrial uptake, oxidation, and ATP synthesis to maintain cellular bioenergetics during stress such as physical activity [22].

Previous reports suggested decreased SM activity [23,24] and mass [25–28] in LAL-D patients, but the role of LAL in the metabolic balance of SM has never been thoroughly investigated. Given the high energy demand of SM, we hypothesized that changes in energy homeostasis caused by the loss of LAL might affect muscle biology. To test our hypothesis, we compared SM from Lal−/− mice with those of their wild-type (Wt) littermates. We found that muscles from Lal−/− mice exhibited morphological and biochemical abnormalities that affected SM phenotype, fiber type distribution, proteomic profiles, and mitochondrial functions, which were associated with compromised exercise performance on a treadmill.

## 2 Materials and Methods

### 2.1 Animals

Male young (8–12 or 12–17 weeks of age) and mature (40–55 weeks of age), as well as female young (19–21 weeks of age) and mature (33–44 weeks of age) Lal−/− mice and their corresponding Wt littermates on a C57BL/6J background were used for the experiments. We analyzed quadriceps (QU), gastrocnemius (GA), tibialis anterior (TA) (higher percentage of fast-twitch glycolytic fibers), and soleus (SO) (higher percentage of slow-twitch oxidative fibers) harvested under fed and 6- or 12-h fasted conditions. Mice were maintained in a clean, temperature-controlled (22 ± 1 °C) environment on a regular light–dark cycle (12-h light, 12-h dark). Animals had unlimited access to chow diet (4 % fat and 19 % protein; Altromin 1324, Lage, Germany). All animal experiments were performed according to the European Directive 2010/63/EU in compliance with national laws and were approved by the Austrian Federal Ministry of Education, Science and Research, Vienna, Austria (2020-0.129.904; 2022-0.121.513; BMWFW-66.010/0081-WF/V/3b/2017).

### 2.2 Histology and immunofluorescence

SM were carefully dissected, mounted on Tissue-Tek® O.C.T™ (Sakura Finetek, Hatfield, PA), and snap frozen in liquid nitrogen-cooled 2-methylbutane for 10–20 s. Samples were stored at −80 °C prior to cryosectioning. SM cryosections were washed with PBS and blocked with 0.05 % TBST (0.05 % Tween-20 in TBS) containing 10 % anti-goat serum. Slides were incubated with monoclonal anti-myosin (MYH7) (#M8421, 1:300; Sigma–Aldrich, St. Louis, MO) or anti-laminin antibodies (#PA1-16730, 1:500, Thermo Scientific, Waltham, MA) in blocking solution overnight at 4 °C. After washing with TBS, sections were incubated with secondary goat anti-rabbit Alexa Fluor-488 (#A-11008, 1:250) and anti-rabbit Alexa Fluor-594 (#A-11012, 1:250, both Thermo Fisher Scientific, Waltham, MA) antibodies in TBST plus anti-goat serum for 1 h at RT, followed by a 10-min incubation with DAPI. Slides were mounted with Dako Fluorescence Mounting Medium (Agilent Technologies, Santa Clara, CA) and visualized using an Olympus BX63 microscope equipped with an Olympus DP73 camera (Olympus, Shinjuku, Japan). The cross-sectional area (CSA) and Feret diameter of myofibers were determined using Fiji software (ImageJ® Version 1.52d; plugin “Muscle morphometry”). The areas of immunofluorescently stained fibers were quantified using ImageJ software (Version 1.53r).

### 2.3 RNA isolation, reverse transcription, and real-time PCR

RNA from SM was isolated using TRIsure™ (Meridian, Memphis, TE), and 0.5–1 μg RNA was reverse transcribed using the High-Capacity cDNA Reverse Transcription Kit (Applied Biosystems, Carlsbad, CA). Real-time PCR was performed with 6 ng of cDNA and the primer sequences listed in Table S1 on a CFX96 Real Time System™ (Bio-Rad Laboratories, Hercules, CA) using GoTaq® qPCR Mastermix (Promega, Madison, WI). Samples were analyzed in duplicate and normalized to *cyclophilin A* expression as the reference gene. Expression profiles and associated statistical parameters were determined by the 2^−ΔΔCt^ method.

### 2.4 Proteasome activity assay

To determine chymotrypsin-like, trypsin-like, and caspase-like activities in SM, we used the Proteasome Activity Fluorometric Assay Kit II (UBPBio, Aurora, CO) according to the manufacturer’s protocol. The standard curve was used to calculate the absolute amounts of released 7-amino-4-methylcoumarin (AMC) fluorescence in each sample.

### 2.5 Amino acid (AA) quantification

Plasma samples (70 μL) from fed and fasted female mice (aged 33–44 weeks) were mixed with 50 μL of 1.5 M perchloric acid, vortexed, and kept at RT for 2 min before adding 1.125 mL of water and 25 μL of 2 M K_2_CO_3_. The tubes were again vortexed, centrifuged at 3,000×*g* for 5 min, and the supernatant was collected and stored at −80 °C until analysis. AA were separated using a high-performance liquid chromatography (HPLC) system equipped with an LC20AD pump (Shimadzu, Kyoto, Japan), an autosampler (Waters 717 plus; Waters, Milford, MA), and a scanning fluorescence detector (Waters 474, Waters, Milford, MA) controlled by LabSolutions software. Chromato-graphic separation was performed using a Supelcosil LC18 3 μm column (150 × 4.6 mm) (Sigma–Aldrich, St. Louis, MO). The autosampler was programmed in addition mode to mix 25 μL of sample with 25 μL of o-phthalaldehyde reagent in the sample loop. The delay time was set to 1 min. A mixture of phase A (0.1 M sodium acetate, methanol, and tetrahydrofuran, pH 7.2) and phase B (methanol) was used as the mobile phase at a flow rate of 1.1 mL/min). The gradient program is shown in Table S2. Fluorescence was measured at an excitation wavelength of 340 nm and an emission wavelength of 455 nm.

### 2.6 Western blotting

SM samples were lysed in RIPA buffer using Precellys (Bertin Instruments, Bretonneux, France). Fifty micrograms of protein were separated by SDS-PAGE, transferred to a PVDF membrane, and incubated with the following anti-mouse antibodies: monoclonal anti-myosin (MYH7) (#M8421, 1:500; Sigma–Aldrich, St. Louis, MO), pAKT (Ser473) (#4051,1:1,000), AKT (#9272, 1:1,000), p4eBP1 (#2855, 1:1,000), and 4eBP1 (#9644, 1:1,000; all from Cell Signaling, Danvers, MA). GAPDH (#2118, 1:1,000; Cell Signaling, Danvers, MA) and α-tubulin (NB100-690, 1:1,000; Novus, Centennial, CO) antibodies were used as loading controls. HRP-conjugated anti-rabbit (#31460, 1:2,500; Thermo Fisher Scientific, Waltham, MA) and anti-mouse (P0260, 1:1,000; Dako, Glostrup, Denmark) were visualized using the Clarity™ Western ECL Substrate Kit on a ChemiDoc™ MP imaging system (both Bio-Rad Laboratories, Hercules, CA). pAkt/AKT and p4eBP1/4eBP1 ratios were estimated by densitometry (ImageJ® Software, Version 1.53r). MYH7 expression was normalized to the expression of GAPDH.

### 2.7 Sample preparation, data acquisition, and metabolomic analysis by nuclear magnetic resonance (NMR)

Snap-frozen GA samples from young male mice were processed as described previously [29]. NMR spectra were processed and analyzed as recently described [30].

### 2.8 Measurement of ATP by mass spectrometry

SM samples from young male mice (~ 25 mg) were transferred to 2 mL Safe-Lock PP-tubes and sonicated in 500 μL 80 % MeOH containing internal standards (670 pmol glutarate, 168 pmol d4-glycocholic acid; Sigma–Aldrich, St. Louis, MO) using a Bioruptor Pico (30 min, 4 °C, 30 s ON/30 s OFF, frequency high; Diagenode, Denville, NJ). After addition of 300 μL ddH_2_O and 900 μL methyl tertbutyl ether (MTBE), samples were incubated for 15 min at 4 °C under shaking. After centrifugation (18,213×*g*, 10 min, 4 °C), 800 μL of the upper phase was removed and replaced with 800 μL of artificial upper phase (MTBE/MeOH/ddH_2_O, 9/4/4, v/v/v). After re-incubation and centrifugation as described above, the complete upper phase was removed, and 800 μL of the lower phase was collected and dried using a SpeedVac (Thermo Fisher Scientific, Waltham, MA). Water-soluble metabolites were resolved in 100 μL of 70 % acetonitrile (ACN)/30 % H_2_O/0.5 mM medronic acid and used for liquid chromatography/mass spectrometry analysis. Tissue residues were dried, solubilized in 0.3 N NaOH (55 °C, ~4 h), and protein content was determined using Pierce™ BCA reagent (Thermo Fisher Scientific, Waltham, MA) according to the manufacturer’s guidelines. For external calibration, ATP concentrations (20 pmol - 200 nmol; Sigma–Aldrich, St. Louis, MO) were prepared in 500 μL of 80 % MeOH containing an internal standard and processed as described above.

Chromatographic separation was performed on a Vanquish UHPLC + system (Thermo Fisher Scientific, Waltham, MA) equipped with an ACQUITY UPLC BEH Amide column (2.1 × 150 mm, 1.7 μm; Waters, Milford, MA) using an 18-min gradient (400 μL/min) of 97 % solvent A (ACN/ddH_2_O, 95/5, v/v; 10 mM NH_4_FA, 10 mM NH_3_) to 65 % solvent B (ddH_2_O/ACN, 95/5, v/v; 20 mM NH_4_FA, 20 mM NH_3_). The column compartment was kept at 40 °C. A QExactive Focus mass spectrometer (Thermo Fisher Scientific, Waltham, MA) equipped with a heated electrospray ionization source (HESI II) was used to detect metabolites in negative data-dependent acquisition mode (*m/z* 60–900). ATP was identified based on the accurate *m/z* of the [M–H]- ion (<5 ppm) and comparison of the retention time and MS2 spectra to a synthetical reference compound (Sigma–Aldrich, St. Louis, MO). Blanks were subtracted from ATP peak areas, normalized to the internal standard, and quantified by comparing ATP/standard ratios to the external calibration curve. ATP concentrations per sample were normalized using the sample wet weight.

### 2.9 2-Deoxy-D-glucose uptake

Six-hours fasted young female mice were injected intraperitoneally with a 10 % 2-deoxy-d-glucose solution in PBS containing 2 μCi [^3^H]-2-deoxy-d-glucose/30 g body weight. Animals were sacrificed 60 min post-injection, and liver and SM were isolated, lyophilized for 48 h, and dry weight was measured. Tissues were then digested in 1 mL of 1 M NaOH overnight at 65 °C, transferred to a scintillation vial containing 8 mL scintillation cocktail, mixed properly, and stored overnight at 4 °C. Radioactivity was determined by liquid scintillation counting and normalized to dry tissue weight.

### 2.10 Lipid extraction and biochemical analysis

Lipid extraction and quantification of TG, total cholesterol (TC), free cholesterol (FC), and CE concentrations were performed as previously described [31].

### 2.11 Analysis of acylcarnitines by mass spectrometry

SM samples from overnight fasted young (8–12 weeks old) male mice were pulverized in liquid nitrogen and 5–15 mg were transferred to 2 mL Safe-Lock PP-tubes. Lipids were extracted according to Matyash et al. [32]. In brief, samples were homogenized with two 6-mm steal beads on a Mixer Mill (Retsch, Haan, Germany; 2 × 10 s, frequency 30/s) in 700 μL MTBE/MeOH (3:1) containing 500 pmol butylated hydroxytoluene, 1 % acetic acid, and 3 pmol palmitoyl-1,2,3,4–^13^C4-l-carnitine (Sigma–Aldrich, St. Louis, MO) as internal standards. Lipids were extracted under constant shaking for 30 min at RT. After addition of 140 μL dH_2_O, samples were vigorously vortexed (3 × 10 s) and centrifuged at 1,000×*g* for 15 min. Thereafter, 500 μL of the upper, organic phase was collected and dried under a stream of nitrogen. Lipids were dissolved in 500 μL MTBE/methanol (3:1) and diluted 1:5 in 2-propanol/methanol/dH_2_O (7:2.5:1) for UHPLC-QqQ analysis. The remaining protein slurry was dried and used for BCA protein determination after lysis in 300 μL of 0.3 N NaOH at 60 °C. Chromatographic separation was performed on a 1290 Infinity II LC system (Agilent, Santa Clara, CA) equipped with a Zorbax RRHD Eclipse Plus C18 column (2.1 × 50 mm, 1.8 μm; Agilent) and a 10-min gradient of 95 % solvent A (H_2_O; 10 mM ammonium acetate, 0.1 % formic acid, 8 μM phosphoric acid) to 100 % solvent B (2-propanol; 10 mM ammonium acetate, 0.1 % formic acid, 8 μM phosphoric acid) at a flow rate of 500 μL/min. The column compartment was kept at 50 °C. Lipids were detected in positive mode using a 6470 triple quadrupole mass spectrometer (Agilent, Santa Clara, CA) equipped with an ESI source. Acylcarnitine species were analyzed by dynamic multiple reaction monitoring ([M+H] + to *m/z* 84.9, CE 28, Fragmentor 164, CAV 5). Data acquisition and processing were performed by MassHunter Data Acquisition software (Version 10.0 SR1, Agilent, Santa Clara, CA) and MassHunter Workstation Quantitative Analysis for QQQ (Version 10.0, Agilent, Santa Clara, CA), respectively. Data were normalized for recovery, extraction, and ionization efficacy by calculating analyte/internal standard ratios (AU) and expressed as AU/μg protein.

### 2.12 Measurement of mitochondrial respiration and fatty acid oxidation (FAO)

Oxygen consumption for estimation of mitochondrial respiration and FA oxidation (FAO) in permeabilized SM fibers was measured with a high-resolution Oxygraph-2k respirometer (Oroboros Instruments, Innsbruck, Austria) as described previously [33]. Briefly, the predominantly oxidative part of GA was separated into small bundles and permeabilized with saponin (50 μg/mL). One to 3 mg of the fibers were transferred to the calibrated respirometer, which contained 2 mL of respiration medium (MiRO6; 110 mM d-sucrose, 60 mM potassium lactobionate, 0.5 mM EGTA, 3 mM MgCl_2_, 20 mM taurine, 10 mM KH_2_PO_4_, 20 mM HEPES, 1 g/L bovine serum albumin, and ~ 280 U/mL catalase) in each chamber.

Substrate/uncoupler/inhibitor (SUIT) protocol 11 was used to examine mitochondrial respiration. The following substrates and inhibitors were added sequentially after the oxygen slope was stable: 10 mM malate followed by 10 mM glutamate (basal respiration), 5 mM ADP (active ADP-stimulated state), 10 μM cytochrome C, 10 mM succinate, 1 μM carbonyl cyanide m-chlorophenylhydrazone (CCCP), 0.5 μM rotenone, and 2.5 μM antimycin. To analyze FAO, we used the SUIT-005 protocol with some modifications. The following substrates and inhibitors were added sequentially after the oxygen slope was stable: 100 μM and 2 mM malate (basal respiration and respiratory stimulation of FAO) (F-pathway), followed by 25 mM ADP, 10 μM cytochrome C, 5 mM pyruvate (respiratory stimulation by simultaneous action of the F-pathway and NADH electron transfer pathway (ETP, N-pathway) together with convergent electron), 10 mM succinate, 1 μM CCCP, 0.5 μM rotenone, and 2.5 μM antimycin. Respiration signals were analyzed using DatLab O2k-6 software.

### 2.13 Electron microscopy

GA was collected from Lal−/− and corresponding control mice perfused with 4 % paraformaldehyde. The muscles were then processed as previously described [31].

### 2.14 Quantification of the number of mitochondria

The concentration of extracted DNA was estimated using Nanodrop (Peqlab, Darmstadt, Germany) and diluted to 10 ng/μL for qPCR amplification to compare the expression of *16S* (mitochondrial gene) with that of hexokinase 2 (*hk2*, nuclear gene). Primer sequences are listed in Table S1. The number of mitochondria was determined from the ratio of mitochondrial (mt) to nuclear (n)DNA as previously described [34].

### 2.15 Measurement of maximum O_2_ consumption (VO_2 max_) and peak effort testing using treadmills

Effort tolerance, peak effort, and maximal oxygen consumption (VO_2 max_) of Wt and Lal−/− mice were studied using a motorized treadmill coupled to a calorimetric unit with gas analyzer (CaloTreadmill, TSE Systems GmbH, Bad Homburg, Germany) as described previously [35]. Briefly, mice were subjected to a ramp running protocol with an initial adaptation velocity of 3 m/min for 60 s, followed by a constant acceleration of 3 m/min without inclination. The exercise session ended at maximal exhaustion, defined as the animal’s inability to maintain running speed despite being in contact with the electrical grid for more than 5 s. VO_2 max_ and run distance were determined at the point at which oxygen uptake reached a plateau during exhaustive exercise. Maximum workload was calculated as the final running distance multiplied by body weight and divided by 1,000.

### 2.16 Sample preparation and processing for proteomics analysis

The “red” deep proximal and medial and the “white” most superficial parts of GA were lysed in 100 mM Tris–HCl (pH 8.5) containing 1 % SDS, 10 mM Tris(2-carboxyethyl)phosphine, and 40 mM chloroacetamide using Bead Mill Max in combination with 2.8 mm ceramic beads (VWR International GmbH, Darmstadt, Germany). Samples were then reduced and alkylated at 95 °C for 10 min and centrifuged at 7,000×*g* and 4 °C for 5 min to remove cell debris. After protein estimation by Pierce™ BCA Protein Assay (Thermo Fisher Scientific, Waltham, MA), 50 μg of each sample was precipitated with acetone, dissolved in 50 mM Tris–HCl (pH 8.5), and digested with Promega Trypsin/LysC Mix (25:1) by overnight shaking at 37 °C. Thereafter, 4 μg of the peptide solution was acidified to a final concentration of 1 % trifluoroacetic acid and desalted using self-made stage-tips with styrenedivinylbenzene - reversed phase sulfonate as material.

### 2.17 Proteome analysis by liquid chromatography-tandem mass spectrometry (LC-MS/MS)

Peptides were separated on the UltiMate™ 3000 RSLCnano Dionex system (ThermoFisher Scientific, Waltham, MA) using an IonOpticks Aurora Series UHPLC C18 column (250 mm × 75 μm, 1.6 μm) (IonOpticks, Fitzroy, Australia) by applying an 86.5 min gradient at a flow rate of 400 nL/min at 40 °C (solvent A: 0.1 % formic acid in water; solvent B: acetonitrile with 0.1 % formic acid; 0–5.5 min: 2 % B; 5.5–25.5 min: 2–10 % B; 25.5–45.5 min: 10–25 % B, 45.5–55.5 min: 25–37 % B, 55.5–65.5 min: 37–80 % B, 65.5–75.5 min: 80 % B; 75.5–76.5 min: 80-2% B; 76.5–86.5 min: 2 % B). The timsTOF Pro mass spectrometer (Bruker Daltonics GmbH, Bremen, Germany) was operated as follows: positive mode, enabled trapped ion mobility spectrometry (TIMS), 100 % duty cycle (ramp 100 ms); source capillary voltage: 1600 V; dry gas flow: 3 L/min, 180 °C; scan mode: data-independent parallel accumulation−serial fragmentation as previously described by Meier [36] using 21 x 25 Th isolation windows, *m/z* 475–1,000; 0 Th overlap between windows. Two and three isolation windows were fragmented per TIMS ramp after the MS1 scan, respectively (overall DIA cycle time: 0.95 s).

### 2.18 LC-MS/MS proteomics data processing, bioinformatics, and statistical analysis

Raw data files were analyzed and proteins were quantified using DIA-NN software (version 1.8.1) [37,38]. The SwissProt *Mus musculus* proteome database in fasta format (containing common contaminants; downloaded on 2021/08/17, 17,219 sequences) was used for a library-free search with false discovery rate (FDR) set to 1 %. Deep learning-based spectra and retention time prediction was enabled, minimum fragment *m*/*z* was set to 200 and maximum fragment *m*/*z* to 1800. N-terminal methionine excision was enabled, and the maximum number of trypsin missed cleavages was set to 2. The minimum peptide length was set to 7 and the maximum to 30 AA. Cysteine carbamidomethylation was used as fixed modification and methionine oxidation as variable modification. DIA-NN optimized the mass accuracy automatically using the first run in the experiment. Data processing using protein group quantities and functional analysis were done with Perseus software version 1.6.15.0, Jupyter Notebook using Python version 3.9 and Cytoscape. Protein intensities were log2-transformed before filtering the data. To avoid exclusion of relevant proteins that were not detected because of low expression in one of the conditions (either Wt or Lal−/−), data were filtered for at least 4 valid values from 5 to 6 samples in at least one group and missing values were replaced by random values from the Gaussian distribution (width of 0.3, downshift of 1.8). Principal component analyses were performed on standardized data (z-scored) and visualized with Jupyter Notebook using the Pandas, Numpy, Matplotlib, Sklearn, Seaborn, and Bioinfokit packages. Two-sample t-tests followed by correction of multiple testing by the permutation-based FDR method were used to identify altered protein groups (S0 = 0.1, FDR <0.01). Enrichment analysis for Gene Ontology Biological Process (GOBP), GO Cellular Component (GOCC), and Reactome pathways was performed using the PANTHER enrichment test for log2-fold changes of proteins [39,40]. Significantly changed pathways are shown in Supplementary Table S3.

### 2.19 Statistical analyses

Statistical analyses and graphs were performed using GraphPad Prism 9 software. Significance was calculated by unpaired Student’s t-test and analysis of variance (one-way ANOVA) followed by Bonferroni post-test. Data are shown as mean ± SD or + SD. The following levels of statistical significance were used: *p < 0.05, **p ≤ 0.01, ***p ≤ 0.001. Bioinformatical and statistical analysis of -omics analyses are described in section 2.18.

## 3 Results

### 3.1 Reduced cross-sectional area and SM mass in Lal −/− mice

**Figure 1:**
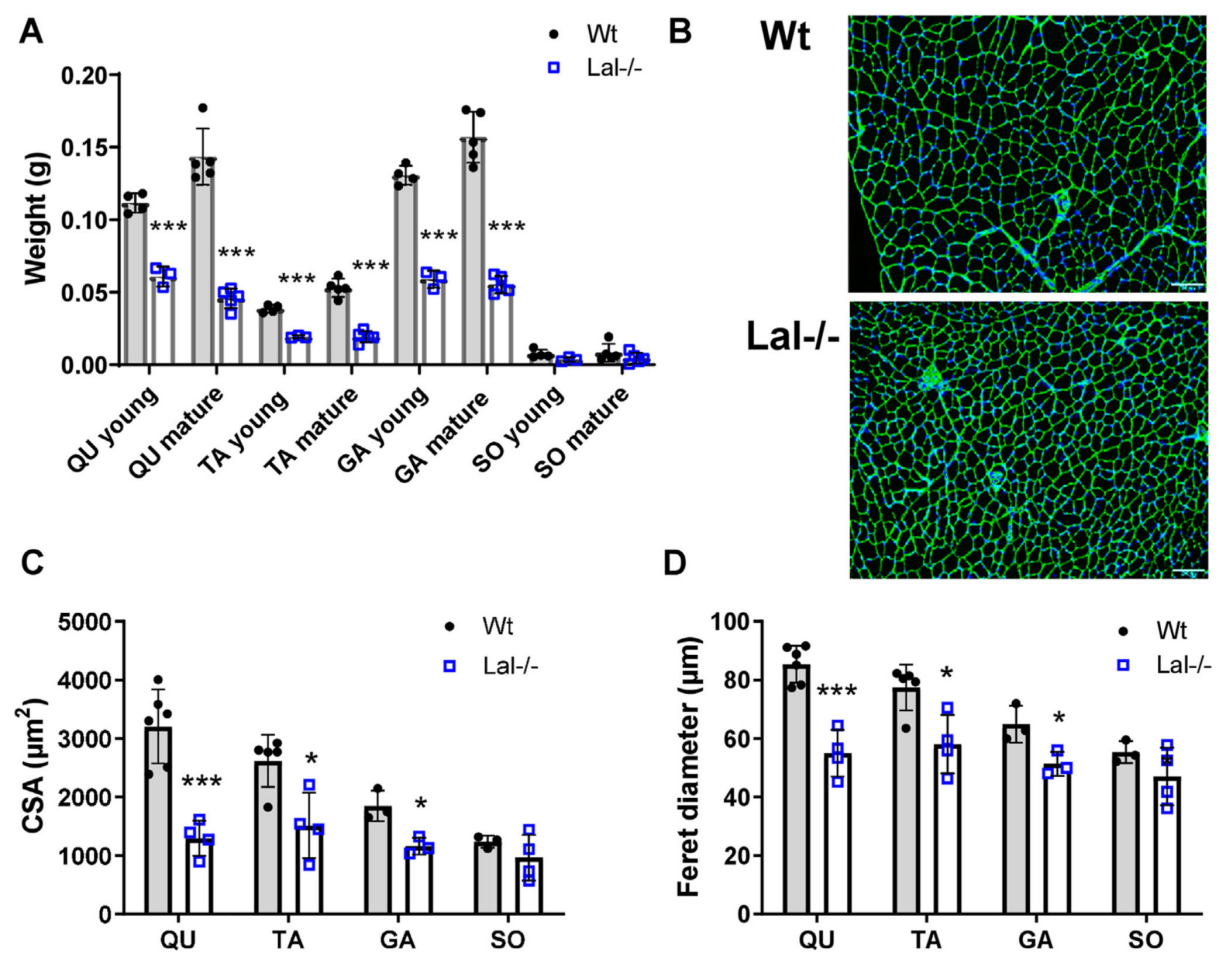
Decreased SM mass, fiber size, and Feret diameter in Lal−/− mice. Four types of SM (quadriceps (QU), tibialis anterior (TA), gastrocnemius (GA), soleus (SO)) were isolated from male Wt and Lal−/− mice aged 15–16 weeks (young) or 40–50 weeks (mature). **(A)** Weight of the SM types. **(B)** Representative images of GA and **(C)** quantification of laminin-stained cross-sectional area (CSA) as well as **(D)** minimum Feret diameter from young male mice (scale bar, 100 μm, 1 to 4 technical replicates from n = 3–5 mice). Data represent mean ± SD. *p < 0.05, ***p ≤ 0.001. Unpaired Student’s t test.

To study the consequences of LAL loss in SM, we isolated gastrocnemius (GA), quadriceps (QU), tibialis anterior (TA), and soleus (SO) and compared the mass between male Wt and Lal−/− mice in the fed (15–16 weeks of age, designated young) and fasted (40–55 weeks of age, designated mature) states. The absolute (Figure 1A) and relative weights (Figs. S1B and C) of QU, TA, and GA were drastically reduced in Lal−/− mice at young and mature ages, whereas the body weights of Lal−/− mice were approx. 30 % lower compared to controls (Fig. S1A). The weight of SO remained unchanged and comparable with those of Wt mice (Figure 1A, S1B,C). Of note, the muscles from Lal−/− mice were paler than the muscles from Wt mice (Fig. S1D). To quantitatively and qualitatively assess muscle fibers and estimate the muscular phenotype, we determined the cross-sectional area (CSA) and minimum Feret diameter of myofibers by quantifying the laminin-stained area with Image J. Consistent with the reduced muscle mass in Lal−/− mice, the mean fiber CSA (Figure 1B,C) and Feret diameter (Figure 1B,D) of QU, TA, and GA were significantly lower in Lal−/− mice compared to their Wt littermates. Examination of the SM sections and use of the “Muscle morphometry” plugin of Image J failed to reveal signs of myopathy, such as centronucleated myofibers, in either genotype. In summary, these findings demonstrate reduced muscle size in Lal−/− mice.

### 3.2 Unaffected protein turnover in SM of Lal−/− mice

**Figure 2:**
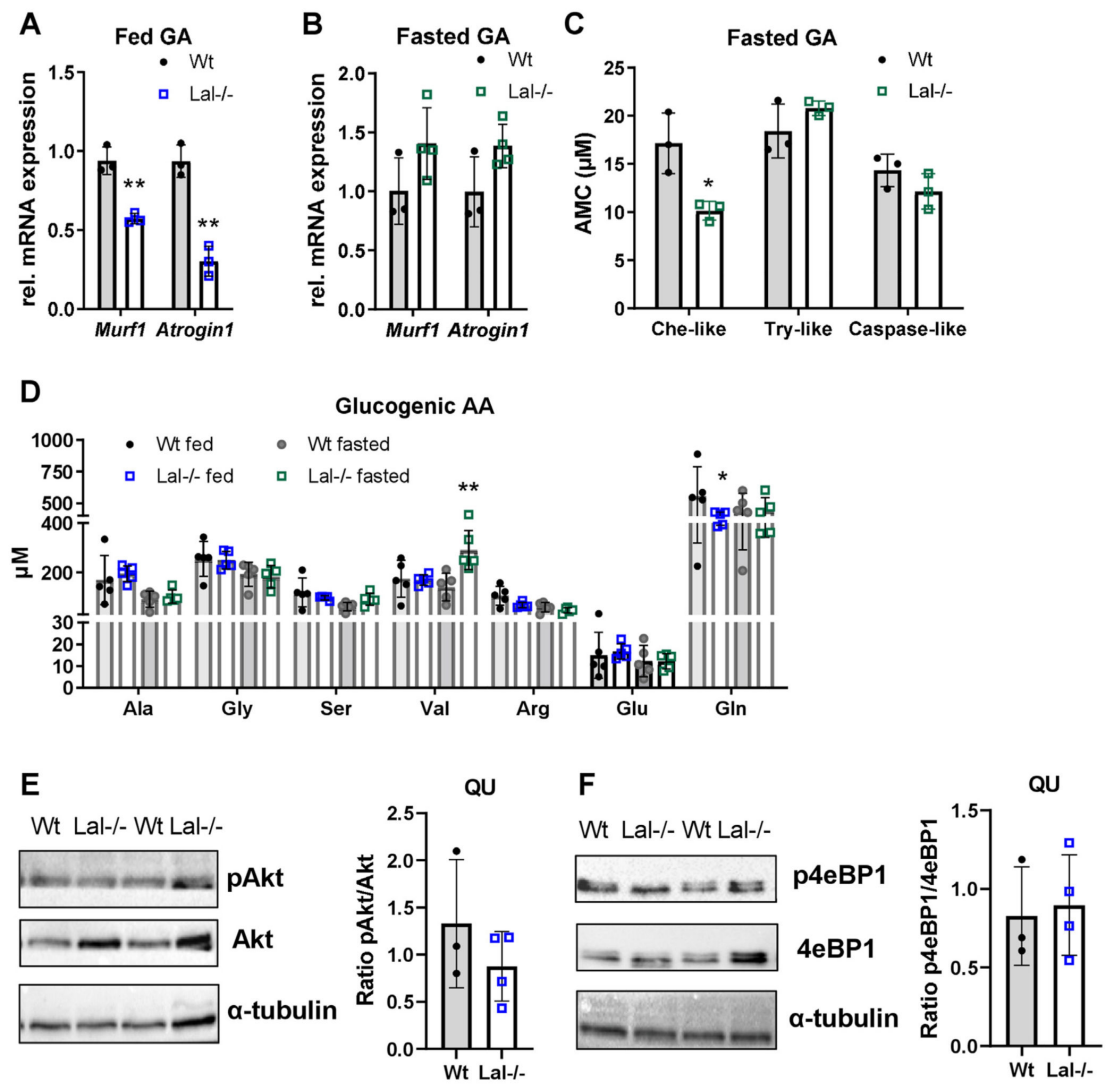
Reduced muscle size in Lal−/− mice is independent of muscle degradation. Relative mRNA expression of genes encoding the proteolysis markers *Murf* and *Atrogin1* in the gastrocnemius (GA) of **(A)** *ad libitum-fed* young male mice (n = 3) and **(B)** young female mice fasted for 12 h (n = 3–4) relative to *cyclophilin A* expression as reference gene. **(C)** Chymotrypsin-like (Che-like), trypsin-like (Try-like), and caspase-like proteasome activities in frozen GA of 6-h fasted young female Wt and Lal−/− mice (n = 3) using 7-amino-4-methylcoumarin (AMC) as fluorescence reference standard. **(D)** Plasma glucogenic amino acid (AA) concentrations quantified using AA separation by high-performance liquid chromatography (HPLC) of mature female *ad libitum-fed* and 12-h fasted Wt and Lal −/− mice (n = 5). **(E, F)** Representative Western blotting experiments of **(E)** pAkt, Akt, **(F)** p4eBP1, and 4eBP1 protein expression in QU and densitometric quantification of **(E)** pAkt/Akt and **(F)** p4eBP1/4eBP1 ratios (n = 4) from young male Lal−/− and Wt mice in the fed state. α-Tubulin expression was used as loading control. Data represent mean ± SD. *p < 0.05, **p ≤ 0.01. **(A–C)** Unpaired Student’s t test. **(D)** One-way ANOVA.

During cold exposure, chow diet-fed Lal−/− mice utilize their muscle AA as an additional energy sources, as indicated by the upregulation of the muscle proteolysis markers *Murf1* and *Atrogin1* in GA [41]. In contrast to the cold-exposed animals, *Murf1* and *Atrogin1* mRNA levels were downregulated in GA (Figure 2A), QU (Fig. S2A), and TA (Fig. S2B) but not in SO (Fig. S2C) of Lal−/− mice in the fed state, whereas no difference was evident in the fasted state in GA (Figure 2B), QU (Fig. S2D), TA (Fig. S2E), and SO (Fig. S2F).

One of the key metabolic mechanisms controlling muscle wasting is the ubiquitin proteasome system [42]. In GA (Figure 2C), QU (Fig. S2G), and TA (Fig. S2H) from Lal−/− mice, chymotrypsin-like activity was reduced, whereas trypsin-like and caspase-like proteasome activities were comparable between the genotypes, arguing against activation of the ubiquitin proteasome system to degrade proteins for energy supply in Lal−/− SM. Analysis of plasma AA concentrations revealed decreased abundance of the glucogenic AA glutamine (Gln) in the fed condition (Figure 2D). Increased concentrations of branched-chain AA such as valine (Val) (Figure 2D), glucogenic/ketogenic isoleucine (Ile) (Fig. S2I), and the ketogenic AA leucine (Leu) (Fig. S2J) in fasted Lal−/− mice indicate minor alterations in AA metabolism, however, may not be associated with muscle fiber degradation in Lal−/− mice.

The IGF-1/Akt/mTOR pathway is a crucial intracellular regulator of muscle mass [43–45]. However, we failed to detect any significant changes in the ratio of pAkt/Akt and p4eBP1/4eBP1 protein expression in Lal−/− QU (Figure 2E,F) and GA (Figs. S2K and L) as well as *Igf1* (Fig. S2M) gene expression in QU and GA from fed Lal−/− and Wt mice.

### 3.3 Loss of LAL impacts SM energy metabolism

**Figure 3:**
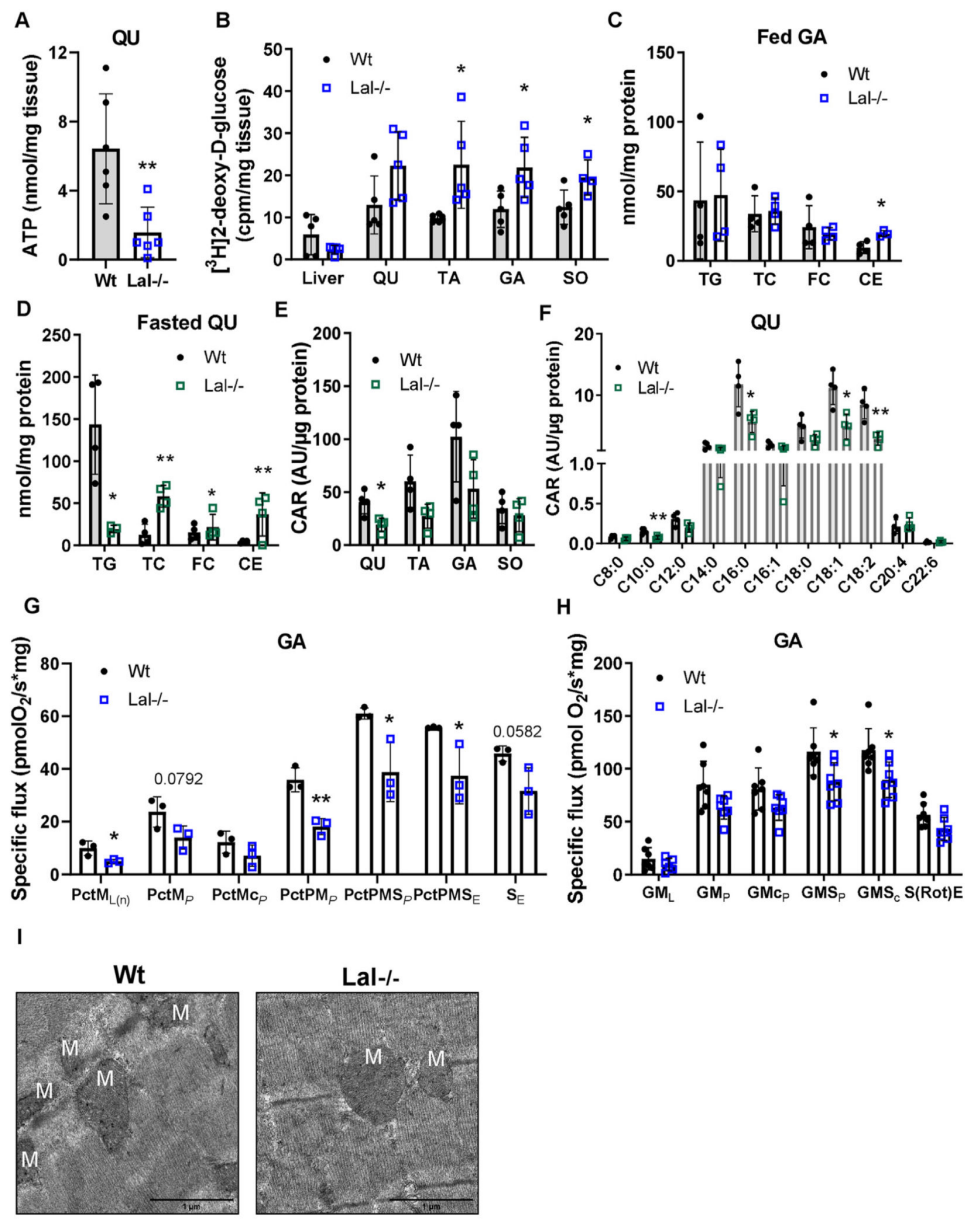
Impaired energy metabolism and mitochondrial respiration in SM of Lal−/− mice. **(A)** ATP concentration in quadriceps (QU) determined by liquid chromatography-tandem mass spectrometry (LC-MS/MS) (young male mice in the fed state; n = 6–7). **(B)** Radioactivity in QU, tibialis anterior (TA), gastrocnemius (GA), soleus (SO), and liver from Wt and Lal−/− female mice after i.p. injection of [^3^H]2-deoxy-D-glucose (n = 5). GA lipid content in **(C)** *ad libitum* fed young and **(D)** 12-h fasted mature male mice (n = 3–4). Quantification of **(E)** total acyl-carnitine (CAR) and **(F)** individual CAR species determined by LC-MS/MS in QU of 12-h fasted young male mice (n = 4). **(G)** Fatty acid oxidation (FAO) in permeabilized fibers isolated from GA of young male Lal−/− and Wt mice. Specific flux was measured at different mitochondrial stages by Oxygraph-2 k high-resolution respirometry. Basal values, respiratory stimulation of FAO (F-pathway) by FA in the presence of malate: PctML(n) (Pct, palmitoyl carnitine; L(n), LEAK (non-phosphorylating resting state); M, malate); OXPHOS capacity: PctM_*P*_ (*P*, saturated ADP), outer mitochondrial membrane integrity assay: PctMc_*P*_ (c, cytochrome C), respiratory stimulation by simultaneous action of the F-pathway and N-pathway (NADH electron transfer-pathway state) with convergent electron flow: PctPM_*P*_ (P, pyruvate); respiratory stimulation by simultaneous action of the F-pathway, N-pathway, and S-pathway (complex II): PctPMS_P_ (S, succinate); uncoupler titration to obtain electron transfer capacity: PctPMS_E_ (E, electron transfer capacity); residual electron transfer capacity: S_E_ (n = 3). **(H)** Mitochondrial respiration in permeabilized fibers isolated from GA of young male Lal−/− and Wt mice. Oxygen consumption was measured at different mitochondrial stages by Oxygraph-2 k high-resolution respirometry. Basal values, respiration at LEAK stage: GML (G, glutamate; M, malate; L, LEAK); OXPHOS capacity P (with saturated [ADP]), active OXPHOS state: GMP (p, pyruvate); outer mitochondrial membrane integrity assay: GMCP (c, cytochrome C); OXPHOS capacity is maximal with convergent electron flow though CI + II: GMSP (S, succinate); uncoupler titration to obtain electron transfer capacity: GMSC; residual oxygen consumption in the ROX state due to oxidative side reactions: S(Rot)E (Rot, rotenone; E, electron transfer capacity) (n = 6). **(I)** Representative electron micrographs of mitochondria of GA from Wt and Lal−/− mice. M indicates mitochondria. Data represent mean ± SD. *p < 0.05, **p ≤ 0.01. Unpaired Student’s t test.

The intramuscular stores of ATP are generally relatively small [46]. The markedly decreased ATP concentrations in QU of Lal−/− mice (Figure 3A) would have to be compensated by activation of metabolic pathways such as phosphocreatine and muscle glycogen degradation followed by anaerobic glycolysis and aerobic carbohydrate and lipid catabolism [46,47]. Creatine and glycogen concentrations were slightly increased in the GA of Lal−/− mice (Figs. S3A and B). Consistent with our previous observation of reduced circulating glucose levels and increased glucose uptake in SM [16], we observed increased [^3^H]2-deoxy-D-glucose uptake in all SM examined (Figure 3B). Surprisingly, glucose uptake was increased not only in SM rich in glycolytic fibers but also in the highly oxidative SO.

Lal−/− mice accumulate lipid-laden lysosomes in ectopic tissues such as the liver, spleen, and small intestine [12,15]. In contrast to these tissues, we found increased CE concentrations in *ad libitum* - fed Lal−/− mice only in GA (Figure 3C), whereas in SO we found only a tendency toward increased CE but elevated concentration of FC (Fig. S3C). Levels of cholesterols in QU and TA (Figs. S3D and E) as well as TG were comparable in all SM (Fig. 3C, S3C-E). In the fasted state, mature Lal−/− mice showed a drastic decrease in TG concentrations in QU (Figure 3D) and trends to decreased concentrations in TA (Figure S3F), GA (Fig. S3G), and SO (Fig. S3H). We additionally observed higher concentrations of CE in QU (Figure 3D), TA (Fig. S3F), and GA (Fig. S3G) in Lal−/− mice compared to the corresponding littermates, apparently due to the increased TC concentrations also in SO (Fig. S3H).

During fasting, lipid oxidation is the predominant source of energy in the resting SM [48]. Since Lal−/− mice suffer from loss of adipose tissue [12] and have reduced circulating TG levels in the fasted state [16], we next analyzed whether FAO was affected in Lal−/− SM.

Despite comparable mRNA expression of genes involved in FA transport and oxidation, with the exception of slightly increased *Cpt1b* expression (Fig. S3K), total acyl-carnitine (Figure 3E) and individual acyl-carnitine concentrations were markedly reduced in QU (Figure 3F), SO (Figure S3I), and TA (Fig. S3J). Respiratory stimulation of the F-pathway at the non-phosphorylating resting state (PctM_L(n)_) and active OXPHOS state (PctM_P_) were reduced in permebealized muscle fibers from Lal−/− mice (Figure 3G). In addition, respiratory stimulation by simultaneous action of F- and N-pathways with convergent electron flow (PctPM_P_), respiratory stimulation by simultaneous action of the F-, N-, succinate pathway (S-, PctPMS_P_), and electron transfer capacity (PctPMS_E_) were decreased (Figure 3G), confirming decreased FA utilization in the SM of fasted Lal−/− mice.

Despite the insufficient FA supply and acyl-CoA in the liver, hepatic mitochondrial function and energy production of Lal−/− mice were unaltered [16]. To assess SM mitochondrial function, we determined the oxygen consumption rate in permeabilized SM fibers by high-resolution respirometry. Loss of LAL resulted in a slight decrease in basal respiration (GM_L_) driven by complex I substrates (glutamate and malate) and the active ADP-stimulated state (GM_P_). Maximal respiration driven by complexes I and II was significantly reduced upon the addition of succinate (GMS_P_) and in the presence of the uncoupler CCCP (GMS_C_) in myofibers from Lal−/− mice, indicating general consequences on mitochondrial coupling (Figure 3H). This result suggests impaired mitochondrial function in Lal−/− SM, regardless of the number or structure of mitochondria, as morphology (Figure 3I) and mtDNA (Fig. S3L) remained unaltered between the genotypes.

### 3.4 Increased expression of oxidative myofiber proteins in Lal−/− mice

**Figure 4:**
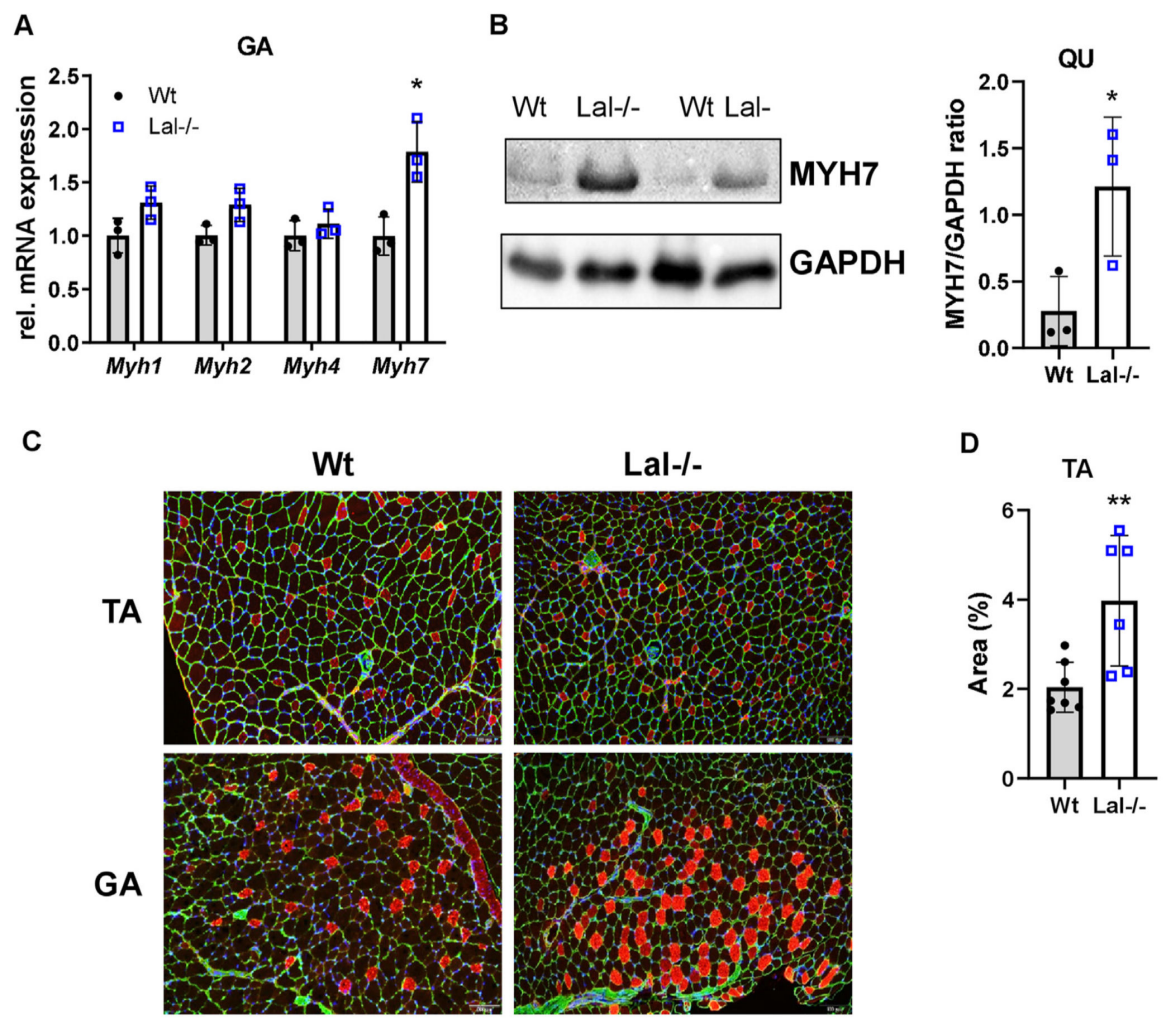
Increased formation of oxidative fibers in skeletal muscles of Lal−/− mice. **(A)** Relative mRNA expression of genes encoding different isoforms of myosin heavy chain in gastrocnemius (GA) of male mice in the fed state (n = 3) relative to *cyclophilin A* expression as reference gene. **(B)** Representative Western blotting experiment of MYH7 protein expression and its densitometric quantification relative to GAPDH (n = 3) in quadriceps (QU) from young 6-h fasted male Lal−/− and Wt mice. GAPDH expression was used as loading control. **(C)** Representative images of immunofluorescence co-staining for MYH7 (red) and laminin (green) in GA and tibialis anterior (TA) cross sections from *ad libitum-fed* Wt and Lal−/− mice. Nuclei are stained with DAPI (blue). Scale bar, 100 μm. **(D)** Percentage of MYH7-stained area quantified using Image J (2–3 technical replicates from 3 mice). Data represent mean ± SD. *p ≤ 0.05. Unpaired Student’s t test.

The distribution and abundance of specific myosin heavy chain (MyHC) isoforms dictate the predominance of oxidative (slow-twitch) or glycolytic (fast-twitch) fibers, thereby affecting the functional properties and metabolic features of SM [49]. Despite increased 2-deoxy-d-glucose uptake (Figure 3A), mRNA expression of *Myh7*, specific for oxidative fibers, was increased in GA (Figure 4A), TA (Fig. S4A), and SO (Fig. S4B) of Lal−/− mice. The markedly increased protein expression of MYH7 in QU (Figure 4B), GA (Figure 4C, S4C), and TA (Figure 4C,D) confirmed the fiber type switch in Lal−/− SM.

### 3.5 Reduced exercise capacity in Lal−/− mice

**Figure 5:**
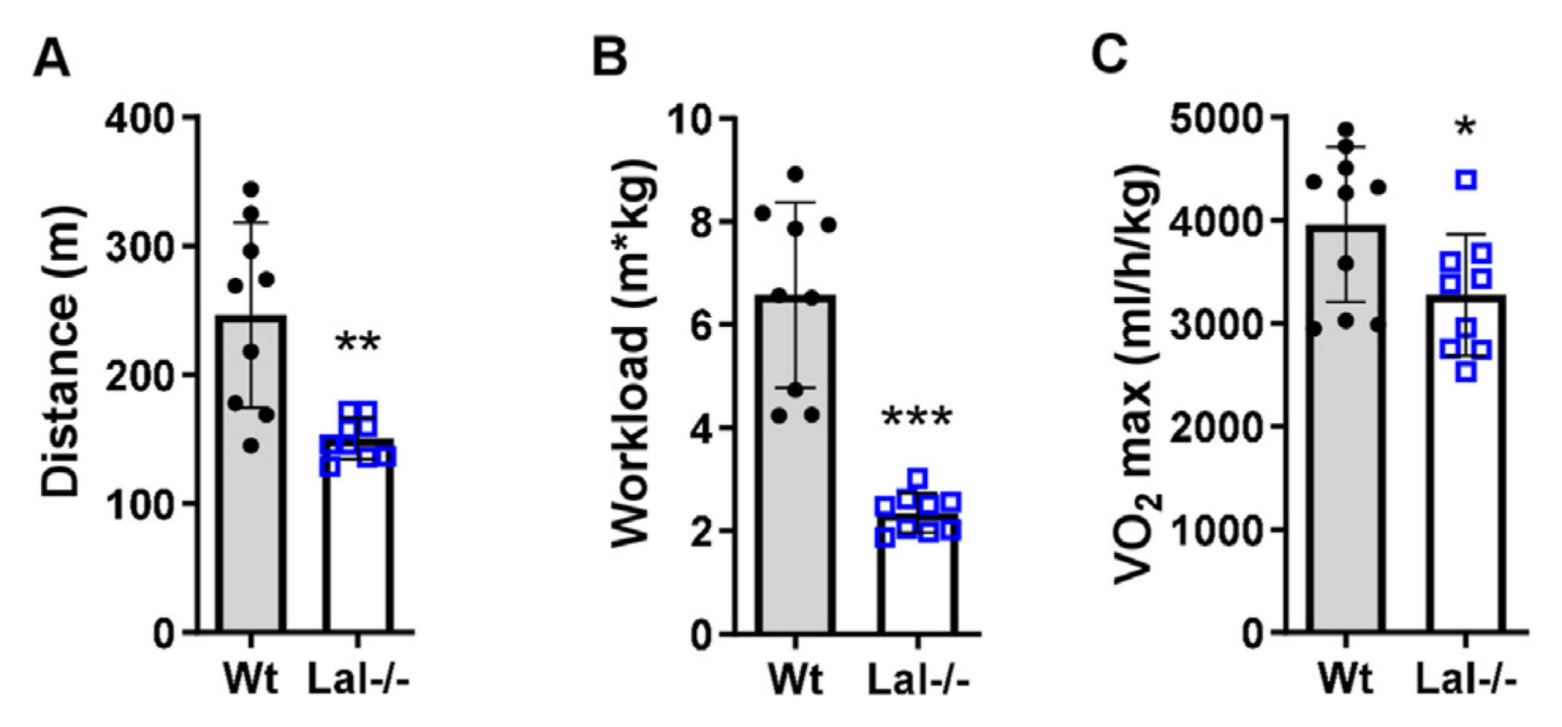
Decreased treadmill exercise performance in Lal−/− mice. Male Wt and Lal−/− mice aged 15–16 weeks were subjected to peak effort testing on a treadmill coupled to indirect calorimetry to determine **(A)** maximal running distance, **(B)** workload, and **(C)** VO_2_ max normalized to body weight. Data show mean ± SD (n = 9–10). *p < 0.05, **p ≤ 0.01, ***p ≤ 0.001 compared to Wt mice. Unpaired Student’s t test.

To investigate whether the observed changes in fiber types translate into reduced physical performance, we performed an exercise tolerance test using the treadmill peak effort test coupled with indirect calorimetry. We found that Lal−/− mice ran significantly shorter distances (Figure 5A) and exhibited a lower total workload (Figure 5B) than Wt mice. Consistent with this observation, Lal−/− mice had lower maximal aerobic capacity (VO_2 max_) compared to Wt mice (Figure 5C). These findings possibly indicate that remodeling of the muscular phenotype translates into impaired exercise capacity *in vivo*.

### 3.6 Altered protein expression patterns in the SM of Lal−/− mice

**Figure 6:**
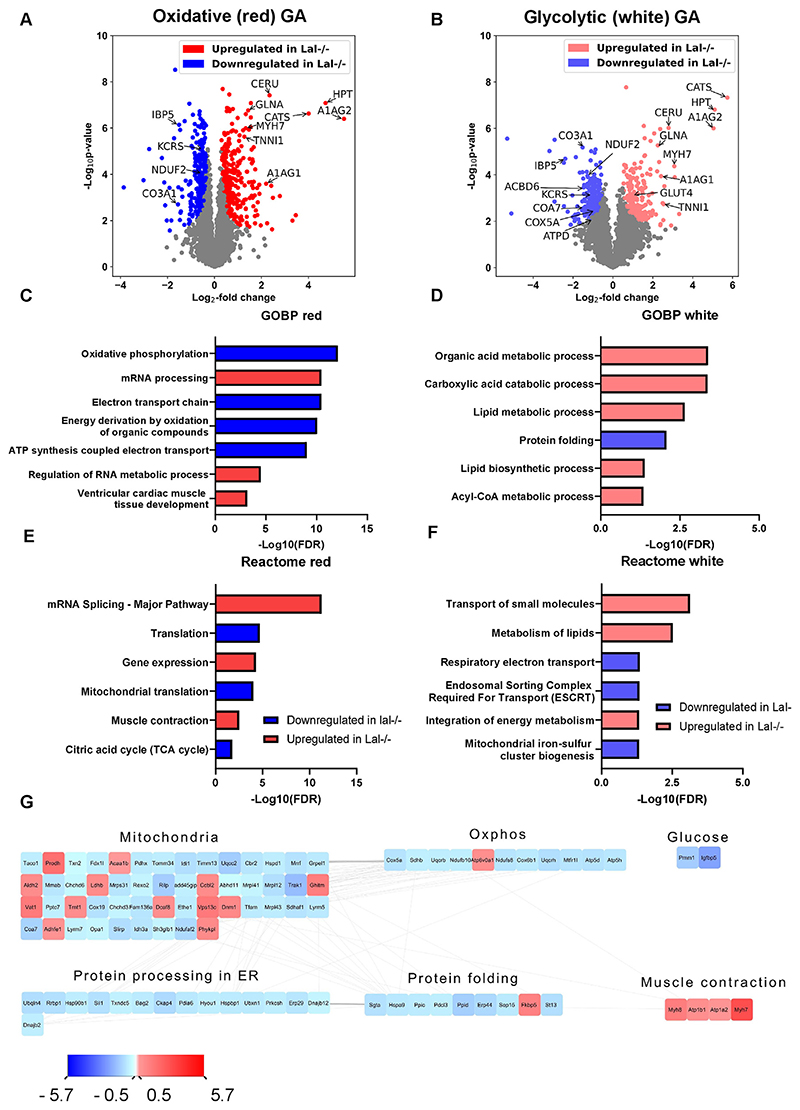
Proteome changes in the oxidative and glycolytic parts of gastrocnemius (GA) in Lal−/− mice. Volcano plots of protein abundance from **(A)** oxidative and **(B)** glycolytic parts of the GA from Wt or Lal−/− young male mice in the fed state (blue, downregulated in Lal−/−; red, upregulated in Lal−/−; grey, unchanged) (n = 5–6) (S0 = 0.1, FDR <0.01). Analyses of the **(C, E)** oxidative (red) and **(D, F, G)** glycolytic (white) parts of GA in Wt and Lal−/− mice. **(C, D)** Downregulated (blue) and upregulated (red) GO terms associated with enriched proteins in SM of Lal−/− mice classified into biological processes (GOBP). **(E, F)** Significantly enriched Reactome pathways in red and white muscle fibers, respectively. **(G)** Protein–protein interaction network represented by the modulation of the significantly changed proteins in the glycolytic (white) part of GA. The interaction network was generated using Cytoscape.

Expression of *Lipa* in SM is low compared with organs with high lipoprotein turnover such as the liver, but is approximately 2-fold higher in the highly oxidative SO than in QU, TA, and GA (Fig. S5). These observed differences between the SM prompted us to gain more insight into the protein abundance between more oxidative and more glycolytic SM parts of Wt and Lal−/− mice. Thus, we divided the GA into “red” (enriched with oxidative fibers) and “white” (enriched with glycolytic ones) parts and performed proteomic analyses. After filtering for a minimum of four valid values out of six in at least one group and imputing missing values, we quantified 3917 proteins in red and 3852 proteins in white muscle fibers. Statistical analysis of the muscle proteome of Lal−/− mice (FDR <0.01, S0 = 0.1) revealed 567 (300 down- and 267 upregulated) and 430 (215 down- and 215 upregulated) significantly changed proteins in red and white muscle fibers, respectively (Table S3). Principal component analysis showed a clear separation between Wt and Lal−/− samples in oxidative (Fig. S6A) and glycolytic (Fig. S6B) parts of GA, with significant up- or downregulation of various proteins in both the red and white parts of the Lal−/− GA, as shown in the volcano plots (Figure 4A,B). The highly upregulated proteins found in both red and white muscle fibers included myosin-7 (MYH7), ceruloplasmin (CERU), cathepsin S (CATS), haptoglobin (HPT), alpha-1-acid glycoprotein 1 and 2 (A1AG1, A1AG2), glutamine synthetase (GLNA), and several types of troponins specific to slow SM (troponin I (TNNI1), troponin C (TNNC1)) (Figure 6A,B; Table S3). Some of the highly downregulated proteins observed in both parts of GA included insulin-like growth factor-binding protein 5 (IBP5), mitochondrial creatine kinase S-type (KCRS), collagen alpha-1(III) chain (CO3A1), and NADH dehydrogenase [ubiquinone] 1 alpha subcomplex assembly factor 2 (NDUF2) (Figure 6A,B; Table S3). A substantial number of the upregulated proteins were related to muscle structure and function, whereas the downregulated proteins were associated with mitochondria and metabolism.

We next performed PANTHER enrichment analysis to identify up- and downregulated GOBP and GOCC terms as well as Reactome pathways (Table S3). Consistent with reduced mitochondrial respiration (Figure 3G), highly significant GOBP terms downregulated in the oxidative part of Lal−/− GA were associated with mitochondrial function and structure, whereas upregulated terms involved RNA processing (Figure 6C). In contrast, the upregulated GOBP terms in the glycolytic part of Lal−/− GA mainly involved various metabolic and catabolic processes (Figure 6D). We also found that protein folding is a significantly downregulated GOBP in Lal−/− white GA (Figure 6D).

We next examined how the loss of LAL affects the expression of proteins annotated to various cellular components. GOCC terms downregulated in the oxidative part of Lal−/− GA included respira-some, mitochondrial membrane, and mitochondrial respiratory chain complex I (Fig. S6C), whereas no GOCC terms were downregulated in the glycolytic part (Fig. S6D). Nucleosome and myosin filament were among the highly significant upregulated GOCC terms in the glycolytic Lal−/− GA part (Fig. S6D). Similarly, in the oxidative part of Lal−/− GA, nuclear protein-containing complex, myosin complex, and spliceosomal complex were upregulated (Fig. S6C).

To investigate metabolic clustering of regulated proteins in more detail, we performed Reactome pathway enrichment analysis. Selected highly upregulated Reactome pathways in the oxidative part of Lal−/− GA were strongly associated with RNA splicing, gene expression, and muscle contraction, whereas the downregulated terms included translation and citric acid cycle (Figure 6E). In the glycolytic part of Lal−/− GA, upregulated Reactome pathways included transport of small molecules, lipid and energy metabolism, whereas the down-regulated terms were related to respiratory electron transport, endosomal sorting complex, and mitochondrial iron-sulfur cluster biogenesis (Figure 6F).

Network analysis using Cytoscape based on protein–protein interactions represented by the significantly differentially expressed proteins revealed comparable interacting clusters in both parts of GA. In particular, we observed clusters formed by several downregulated proteins that play a role in oxidative phosphorylation and mitochondria (Figure 6G and S6E), consistent with reduced ATP concentrations (Figure 3A) and the mitochondrial dysfunction in Lal−/− SM determined by high-resolution respirometry (Figure 3G). Another group of downregulated proteins was clustered in protein processing in the ER and protein folding, whereas upregulated proteins formed clusters related to muscle contraction and muscle development (Figure 6G and S6E). This finding further confirmed the dysfunction of SM mitochondria and fiber switch in Lal−/− mice.

## 4 Discussion

The impact of LAL-D on the pathophysiology of SM is still unclear, although decreased muscle size and muscle weakness were reported as characteristic features of LAL-D patients [23–26,28]. Compromised lipid homeostasis in Lal−/− mice, arising from impaired hepatic and intestinal lipid metabolism as well as lipodystrophy, leads to increased glucose consumption despite unaltered insulin levels, which ultimately results in reduced glucose concentrations in plasma and liver [16]. Since SM require a considerable amount of energy to function efficiently, alterations in lipid and/or glucose metabolism may influence the metabolic state of the muscle and thus the proper functionality of the organ. We therefore aimed to characterize muscle structure, function, and metabolism in an animal model of LAL-D. Our findings demonstrate that the phenotype and metabolism of Lal−/− SM are substantially impaired most likely due to an energy deficit and impaired energy metabolism associated with mitochondrial dysfunction.

Muscles from Lal−/− mice are smaller and exhibit diminished myofiber CSA and Feret diameter. However, preliminary data from our laboratory failed to reveal any differences in SM mass (QU) between 2-week-old Lal−/− and Wt mice or any alterations in muscle differentiation and myogenesis marker genes (data not presented), despite the crucial role of LAL during early development [31]. Thus, we assumed that the reduced SM mass and size worsened with age in Lal−/− mice, reflecting either growth retardation [50] or progressive loss of SM mass due to muscle proteolysis [51]. Plasma AA could serve as a substrate for the translation of muscle proteins [52]. The concentrations of glucogenic AA, which were expected to be increased during starvation due to muscle proteolysis, especially in mature mice, were unchanged or even reduced (Gln and Arg) in Lal−/− compared to Wt mice. Elevated concentrations of the branched-chain AA Leu, Ile, and Val in fasted Lal−/− mice could meet the elevated body demand for Ala and Gln due to increased muscle proteolysis or reduced muscle protein synthesis [53,54]. However, unaltered proteasomal activity and expression of muscle proteolysis markers in the SM of fasted Lal−/− mice argued against muscle wasting being responsible for the smaller size and mass of Lal−/− SM.

PI3K/Akt, activated by either insulin or IGF-1, is critical for controlling protein production in SM [43,44,55]. Other key regulatory proteins involved in translation such as 4E-BP-1 are downstream targets of the mammalian target of rapamycin (mTOR) kinase, which is phosphorylated and triggered by activation of Akt [45,56]. Despite unaltered expression of these markers, translation itself was one of the significantly downregulated terms revealed in Reactome pathways in red muscle fibers, suggesting altered protein synthesis by various signaling pathways, e.g. the nuclear factor kappa B (NF-κB)-depen- dent pathway [45]. Of note, Lal−/− mice suffer from systemic inflammation, as demonstrated by elevated levels of pro-inflammatory cytokines and macrophage infiltration in the liver, spleen, lung, and small intestine [12,15,57,58]. However, despite unaltered *Igf1 and Igf1r* (data not shown) expression, IGFBP5, which generally enhances the effect of IGF-1, was strongly downregulated in both parts of GA. The *Igfbp5* gene encoding IGFBP5 is low expressed during fasting, cachexia, diabetes, and other conditions that cause skeletal muscle atrophy [59]. Recent results also suggested that IGFBP5 modulates the action of autocrine IGF-2 in promoting myogenic differentiation [60], however, they contradict the finding that overexpression of IGFBP-5 under a constitutive promoter impairs myogenic differentiation [61,62]. Nevertheless, a decrease in IGFBP5 expression was described upon muscle denervation or unloading [63], which is in line with decreased locomotor activity [41] and physical capacity in Lal−/− mice during the treadmill peak performance test. It is important to mention that cardiac abnormalities have not been described in Lal−/− mice. Whether the pathophysiological phenotype of Lal−/− lungs [57] contributes to physical performance of Lal−/− mice is currently unknown.

Lal−/− mice display reduced muscle ATP concentrations, resulting in compensatory activation of various metabolic pathways, including increased glucose uptake and degradation of phosphocreatine, glycogen, and lactate [16]. While TG levels remain unchanged in Lal−/− mice in the fed state, fasting leads to reduced TG concentrations in different muscles. The significant decrease in total acyl-carnitine levels and the confirmed reduced FA utilization further underscore the complex metabolic dysregulations in Lal−/− mice that affect both energy production and substrate utilization.The distribution and abundance of specific MyHC isoforms dictate the predominance of “red” oxidative (slow-twitch) or “white” glycolytic (fast-twitch) fibers, thereby influencing the functional properties and metabolic features of the SM. Myofibers of vertebrate SM differ in their contractile properties, mitochondrial density, and metabolic characteristics, with different SM exhibiting a combination of various fibers. Slow-twitch fibers are characterized by the presence of type I MyHC expression and high mitochondrial density, which is predominantly associated with an oxidative metabolism. Fast-twitch fibers express type II MyHCs sub-divided into IIa, IIx, and IIb [49]. Thus, QU, TA, and GA are mainly composed of MyHCIIb (fast glycolytic fibers) but in varying proportions, whereas SO has a combination of MyHCI and MyHCIIa [64]. Changes in muscle size may also be associated with a switch in muscle fiber types, since slow (type I) fibers have a smaller CSA than fast (type II) fibers, which was termed the “muscle fiber type – fiber size paradox” [65]. When we analyzed gene expression and proteome signature changes, MYH7 and other specific slow oxidative muscle markers such as TNNC1 and TNNI1 were upregulated in GA of Lal−/− mice, suggesting that loss of LAL leads to a switch from fast type II to slow type I myofibers with reduced CSA and Feret diameter. However, the mechanism underlying the increased expression of proteins specific to type I fibers remains unclear, especially since glucose consumption was elevated in SM of Lal−/− mice. SM with fast-twitch glycolytic fibers are more sensitive to energy substrate deprivation than slow-twitch oxidative fibers under atrophic conditions [66]. Thus, Lal−/−SM may adapt to the dysfunctional whole-body energy homeostasis, as slow fibers work sufficiently but consume less ATP with lower power utilization than fast fibers [67].

An increased number of oxidative fibers should be associated with more mitochondria, contributing to enhanced cellular respiration [68]. However, we observed decreased oxidative capacity in permeabilized fibers isolated from Lal−/− GA, indicating mitochondrial dysfunction in Lal−/− mice, although the number and morphology of mitochondria remained unchanged. Proteomic analyses confirmed that pathways associated with oxidative phosphorylation, electron transport chain, and ATP synthesis were the most dysregulated biological processes in the red but also the white part of Lal−/− GA. Down-regulated proteins included NDUF, SDH, UQCR, and ATP5 family members representing complexes I, II, III, and V, respectively, which were previously described to be strongly reduced in sarcopenic muscles [69]. ATP plays an essential role in preventing protein aggregation and is a major energy source required for most energy-dependent cellular functions such as protein synthesis (about 30 % of available ATP [56]), folding, translocation to the ER, and protein degradation [70]. Thus, insufficient ATP supply for protein processing may also explain the reduced muscle size in Lal−/− mice. It is worth noting that the downregulated GOBP terms and protein interaction network generated by Cytoscape confirmed impaired protein folding in both the red and white parts of GA.

Interestingly, among the most downregulated proteins, we found major urinary protein 11 (MUP11) in both the oxidative and glycolytic parts of Lal−/− GA, and MUP3 in addition in the red part. The SM was reported to be one of the major metabolic target tissues of MUP1, a close homolog of MUP3 [71]. Low concentrations of circulating MUP1 contribute to the metabolic dysregulation in obese and diabetic mice, which was markedly ameliorated by MUP1 replenishment by increasing the expression of genes involved in mitochondrial biogenesis and enhancing mitochondrial oxidative capacity, predominantly in SM [71]. Our findings may indicate a potential role of other MUPs as potential regulators in the diminished mitochondrial function in Lal−/− mice.

Loss of LAL is associated with ectopic accumulation of lipids, especially CE [12,16,58,72,73], also in SM [74]. Cholesterol accumulation may contribute to mitochondrial dysfunction, including reduced respiration and decreased ATP production [75,76] in Lal−/− SM. The previously described rapid development of fatigue and the reduction in ATP turnover of the SM [77] is consistent with the Lal−/− SM mitochondrial dysfunction and the reduction in ATP concentration. The decreased mitochondrial respiration may also result from an overall reduction in the import of components of the mitochondrial oxidative phosphorylation system and/or substrate transporters throughout the body as a consequence of the inaccessibility of lipids, the absence of white adipose tissue, and the rapid glucose consumption in various organs of Lal−/− mice.

## 5 Conclusion

Taken together, our data provide conclusive evidence that whole-body loss of LAL affects the phenotype and, most probably, functions of SM, due to insufficient ATP production associated with dysfunctional mitochondria and impaired energy metabolism. The described alterations result in SM fiber switch, and Lal−/− mice may serve as a model to study the complex molecular mechanisms of muscle remodeling under conditions of impaired lipid metabolism. However, it is still elusive whether the reduction in muscle mass and increased muscle fatigue are attributable to the global loss of LAL activity in SM or to the systemic loss of the enzyme, which mainly affects the liver, small intestine, and adipose tissue and is associated with severe macrophage infiltration and systemic inflammation.

## Supplementary Material

Fig. S1-S6, Table S1

Table S3

## Data Availability

The mass spectrometry proteomics datasets have been deposited to the ProteomeXchange Consortium via the PRIDE partner repository [78] with the dataset identifier PXD045665 reviewer_pxd045665@ebi.ac.uk. All other data will be made available upon request.

## Data Availability

Data will be made available on request.

## Abbreviations

AA: amino acid
CCCP: carbonyl cyanide m-chlorophenylhydrazone
CE: cholesteryl ester
CSA: cross-sectional area
ETP: electron transfer pathway
FA: fatty acid
FAO: fatty acid oxidation
FC: free cholesterol
FDR: false discovery rate
GA: gastrocnemius
GOBP: gene ontology biological process
GOCC: gene ontology cellular component
LAL: lysosomal acid lipase
Lal−/−: Lal knockout
LAL-D: LAL deficiency
MyHC: myosin heavy chain
QU: quadriceps
SM: skeletal muscle
SO: soleus
TA: tibialis anterior
TC: total cholesterol
TFEB: transcription factor EB
TG: triacylglycerol
Wt: wild-type
