# Supplementary material for: Loss of lysosomal acid lipase results in mitochondrial dysfunction and fiber switch in skeletal muscles of mice
Source: Mol Metab. Author manuscript; Available in PMC 2024 Jan 15. (PMC7615526; doi:10.1016/j.molmet.2023.101869)
Supplement: Fig. S1-S6, Table S1 [file EMS193249-supplement-Fig__S1_S6__Table_S1.pdf]

Supplementary Figures and Tables

Loss of lysosomal acid lipase results in mitochondrial dysfunction and fiber switch in skeletal muscles of mice

Alena Akhmetshina, Valentina Bianco, Ivan Bradić, Melanie Korbelius, Anita Pirchheim, Katharina B. Kuentzel, Thomas O. Eichmann, Helga Hinteregger, Dagmar Kolb, Hansjoerg Habisch, Laura Liesinger, Tobias Madl, Wolfgang Sattler, Branislav Radović, Simon Sedej, Ruth Birner-Gruenberger, Nemanja Vujić, and Dagmar Kratky

Figure S1

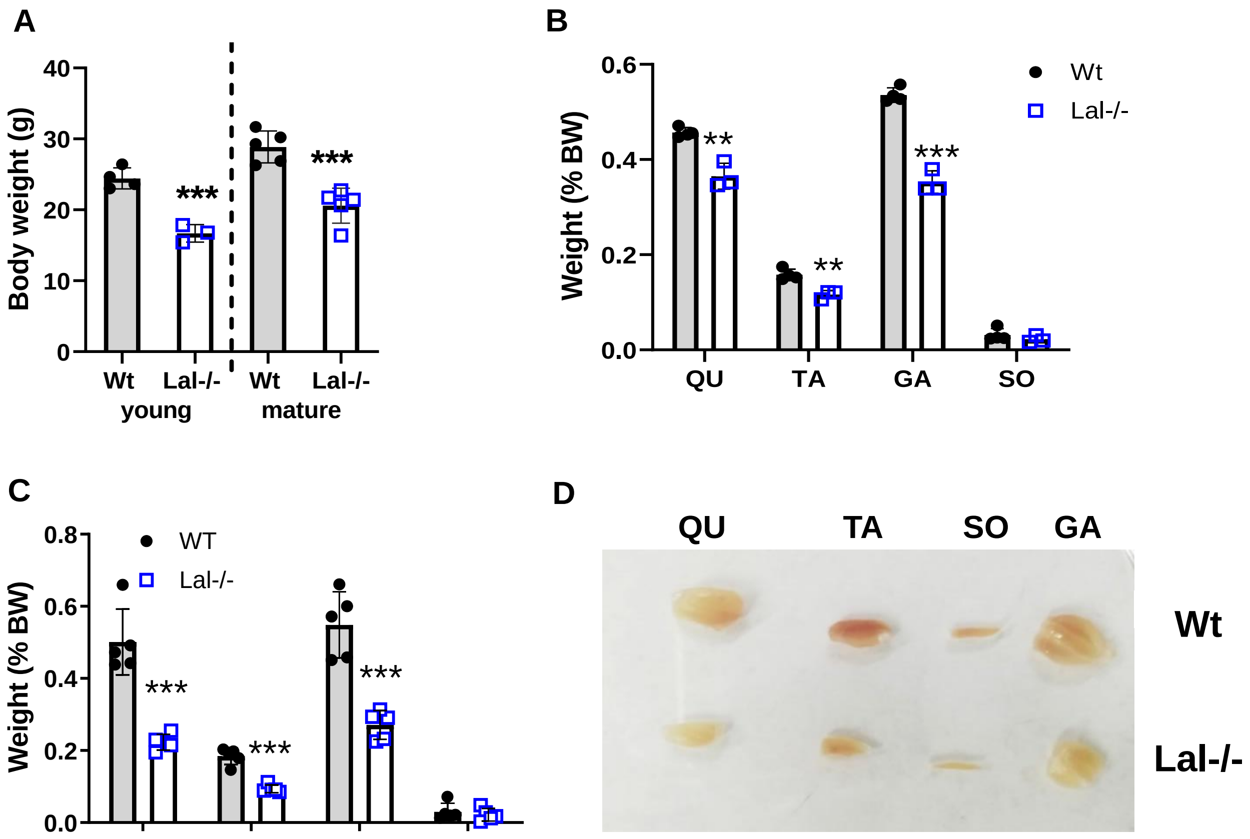

Figure S1. Decreased skeletal muscle (SM) mass in Lal-/- mice.

(A) Body weight of young male mice in the fed state (n=3-4) and mature male mice fasted for 12 h (n=5).Mass of quadriceps (QU), tibialis anterior (TA), gastrocnemius (GA), and soleus (SO) as % of body weight from **(B)** young male mice in the fed state (n=3-4) and **(C)** mature male mice fasted for 12 h (n=5). **(D)** Representative images of isolated SMs form young fed male mice. Data represent mean ± SD **p ≤ 0.01, ***p ≤ 0.001. Unpaired Student's t test.

Figure S2

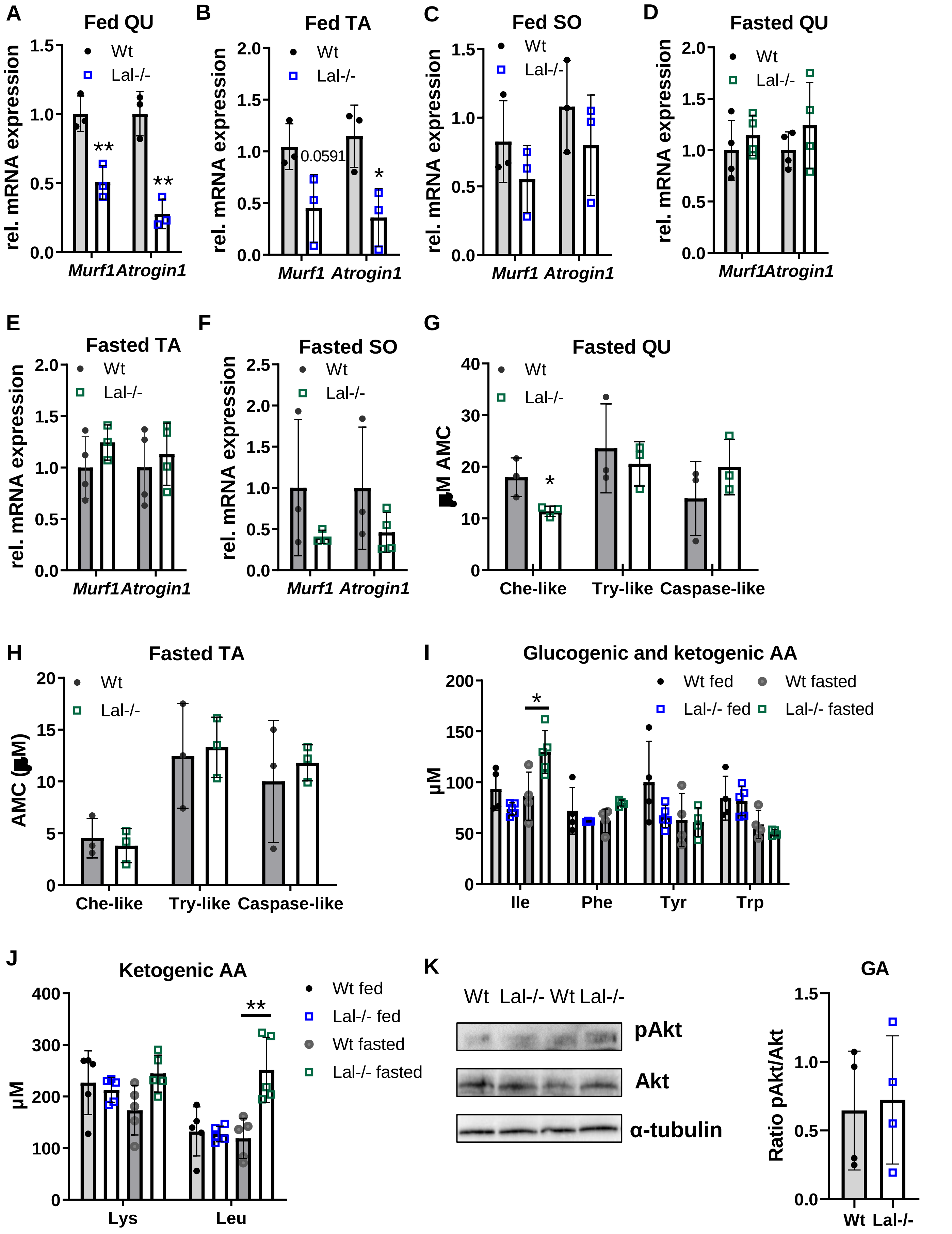
A
Fed QU
rel. mRNA expression
Wt
Lal-/-
**
**
Murf1
Atrogin1
B
Fed TA
0.0591
*
C
Fed SO
D
Fasted QU
E
Fasted TA
F
Fasted SO
G
Fasted QU
µM AMC
*
Che-like
Try-like
Caspase-like
H
Fasted TA
AMC (µM)
I
Glucogenic and ketogenic AA
Wt fed
Wt fasted
Lal-/- fed
Lal-/- fasted
µM
Ile
Phe
Tyr
Trp
J
Ketogenic AA
**
Lys
Leu
K
Wt Lal-/- Wt Lal-/-
pAkt
Akt
α-tubulin
GA
Ratio pAkt/Akt
Wt
Lal-/-

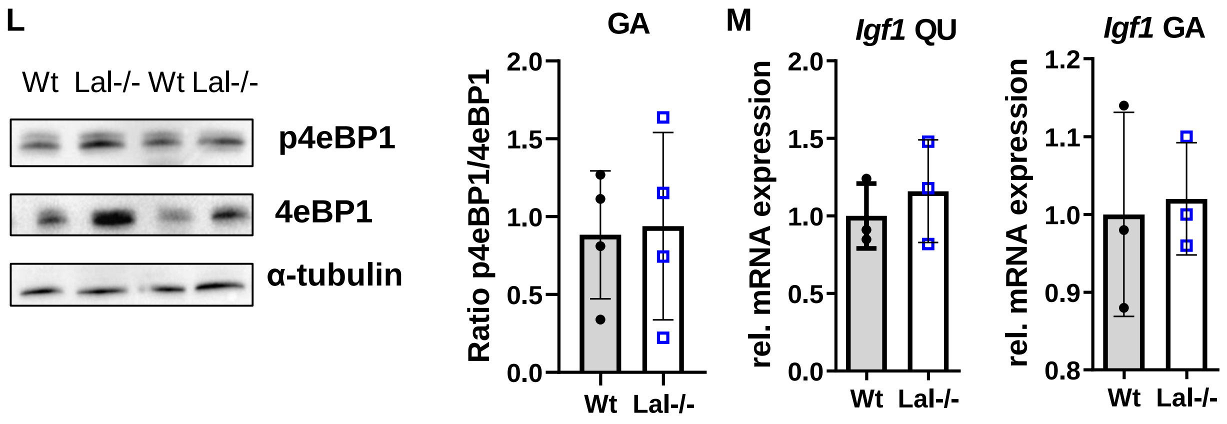

Figure S2. Reduced muscle size in Lal-/- mice is independent of proteolysis markers.

Relative mRNA expression of genes encoding the muscle proteolysis markers *Murf* and *Atrogin1* in **(A)** quadriceps (QU), **(B)** tibialis anterior (TA), and **(C)** soleus (SO) of young male mice (fed state) (n=3) and **(D)** QU, **(E)** TA, and **(F)** SO of young female mice (fasted state) (n=3-4). **(G, H)** Chymotrypsin-like (Che-like), trypsin-like (Try-like), and caspase-like proteasome activities in frozen QU **(G)** and TA **(H)** of young female 12-h fasted Wt and Lal-/- mice (n=3) were calculated using 7-amino-4-methylcoumarin (AMC) as fluorescence reference standard. **(I, J)** Plasma amino acid (AA) concentrations quantified using AA separation by high-performance liquid chromatography (HPLC) of mature female fed and 12-h fasted Wt and Lal−/− mice (n=4-5). **(K, L)** Representative Western blotting experiments of **(K)** pAkt, Akt, **(L)** p4eBP1, and 4eBP1 protein expression in GA and densitometric quantification of **(K)** pAkt/Akt and **(L)** p4eBP1/4eBP1 ratios (n=4) from young male Lal-/- and Wt mice in the fed state. α-Tubulin expression was used as loading control. **(M)** Relative mRNA expression of *Igf1* in QU and GA of young fed male Wt and Lal-/- mice (n=3). Data represent mean ± SD. *p < 0.05, **p ≤ 0.01. **(A-H, K-M)** Unpaired Student's t test. **(I,J)** One-way ANOVA.

Figure S3

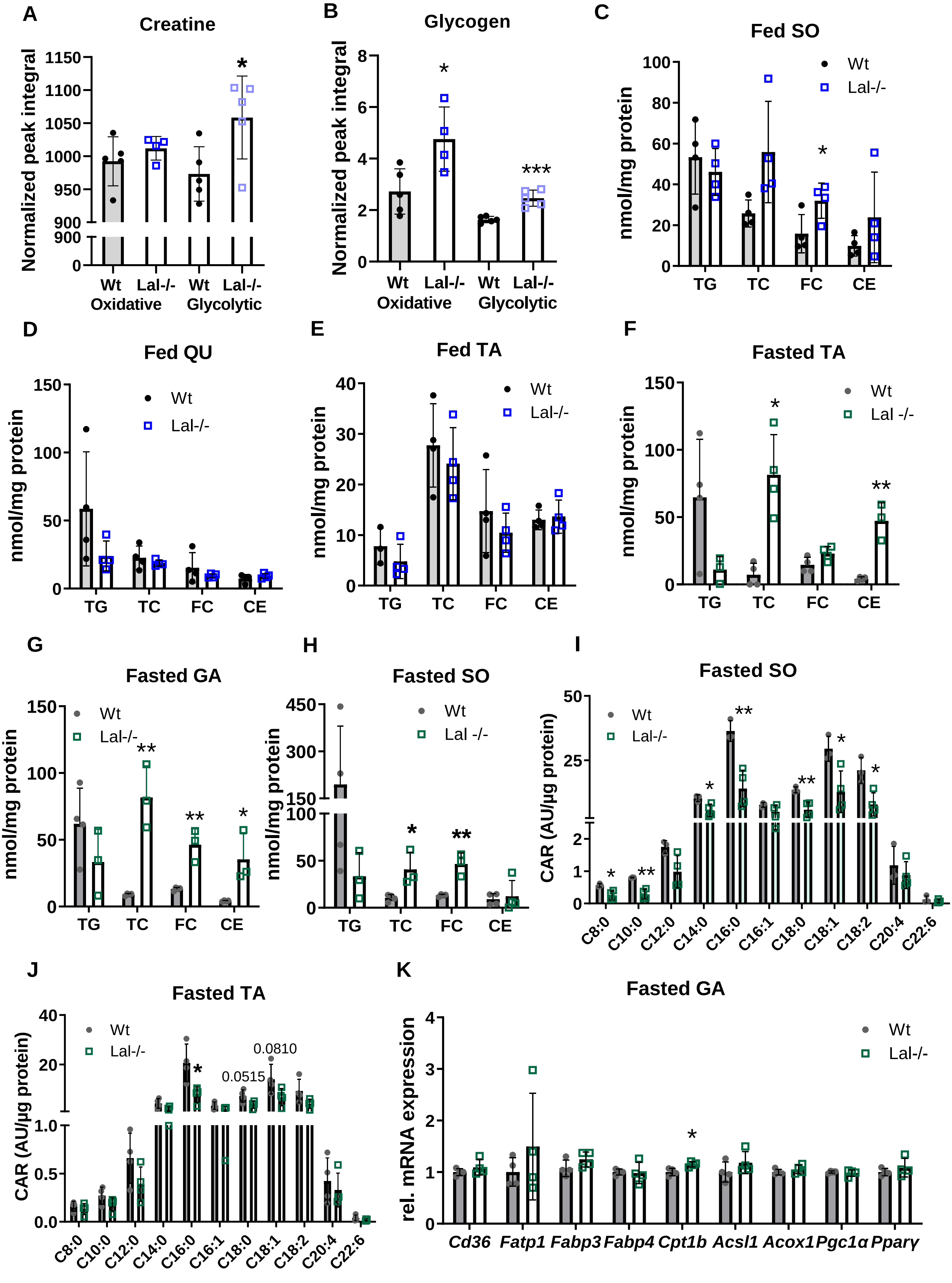

A
Creatine
Normalized peak integral
Wt
Lal-/-
Oxidative
Glycolytic
B
Glycogen
C
Fed SO
nmol/mg protein
TG
TC
FC
CE
D
Fed QU
E
Fed TA
F
Fasted TA
Lal -/-
G
Fasted GA
H
Fasted SO
I
Fasted SO
CAR (AU/μg protein)
C8:0
C10:0
C12:0
C14:0
C16:0
C16:1
C18:0
C18:1
C18:2
C20:4
C22:6
J
Fasted TA
0.0515
0.0810
K
Fasted GA
rel. mRNA expression
Cd36
Fatp1
Fabp3
Fabp4
Cpt1b
Acsl1
Acox1
Pgc1α
Pparγ

L

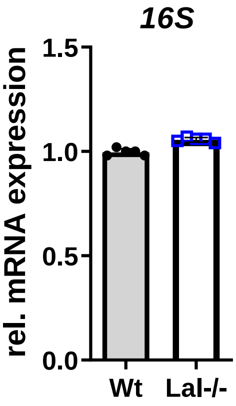

Figure S3. Altered metabolism in SMs of Lal-/- mice.

(A) Creatine and **(B)** glycogen levels determined by nuclear magnetic resonance (NMR) of young fed male mice in gastrocnemius (GA) as normalized peak integral. Biochemical quantification of triacylglycerol (TG), total cholesterol (TC), free cholesterol (FC), and cholesteryl ester (CE) concentrations from **(C-E)** *ab libitum*-fed and **(F-H)** 12-h fasted mature male mice in **(C,H)** soleus (SO), **(D)** quadriceps (QU), **(E,F)** tibialis anterior (TA), and **(G)** GA (n=3-4). Quantification of individual acyl-carnitine (CAR) species determined by liquid chromatography/mass spectrometry in **(I)** SO and **(J)** TA of 12-h fasted young male mice (n = 4). **(K)** Relative mRNA expression of genes involved in fatty acid oxidation in SMs in GA of 12-h fasted young female Wt and Lal-/- mice (n=3-4). **(L)** Mitochondrial DNA copy number in GA of fed young male Lal-/- and Wt mice (n=5). **(A-E, I-M)** Data represent mean ± SD or **(F-H)** mean + SD. *p < 0.05, **p ≤ 0.01 Unpaired Student's t test.

Figure S4

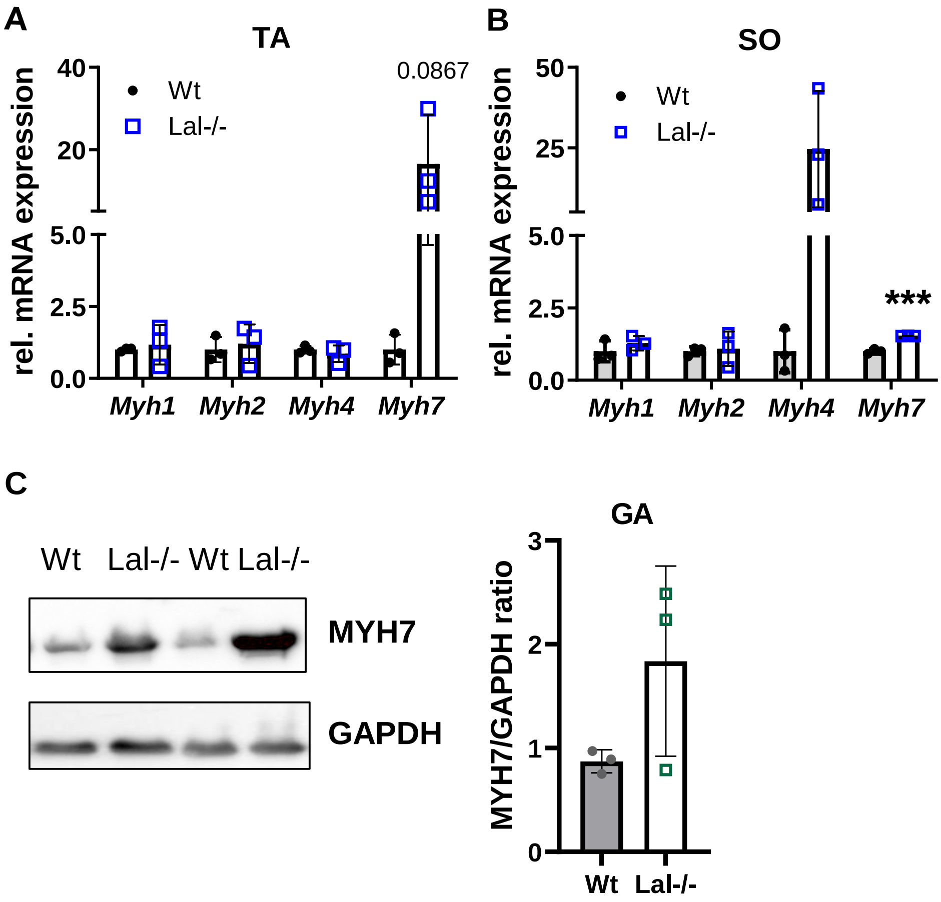

Figure S4. Increased expression of myosin heavy chain specific for oxidative fibers.

Relative mRNA expression of genes encoding different isoforms of myosin heavy chain in **(A)** tibialis anterior (TA) and **(B)** soleus (SO) of male mice in the fed state (n=3) relative to *cyclophilin A* expression as reference gene. **(C)** Representative Western blotting experiment of MYH7 protein expression and its densitometric quantification relative to GAPDH (n=3) in gastrocnemius (GA) from 6-h fasted young male Lal-/- and Wt mice. Data represent mean ± SD. ***$p \leq 0.001$. Unpaired Student's t test.

Figure S5

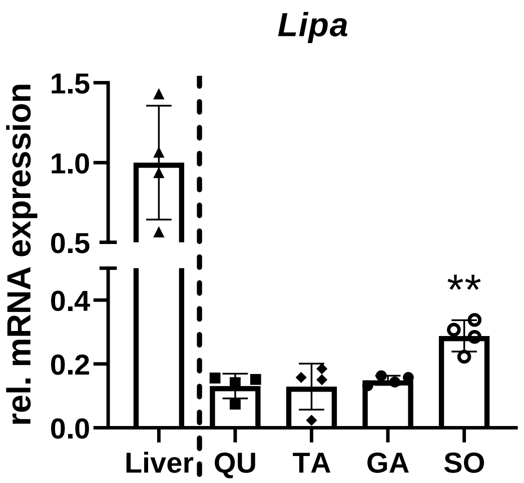

Figure S5. Higher *Lipa* mRNA expression in the highly oxidative SO.

Relative mRNA expression of *Lipa* in liver, quadriceps (QU), tibialis anterior (TA), gastrocnemius (GA), and soleus (SO) of mature Wt mice (n=4). Data represent mean ± SD. **p ≤ 0.01. One-way ANOVA between skeletal muscles; expression in liver was plotted as reference.

Figure S6

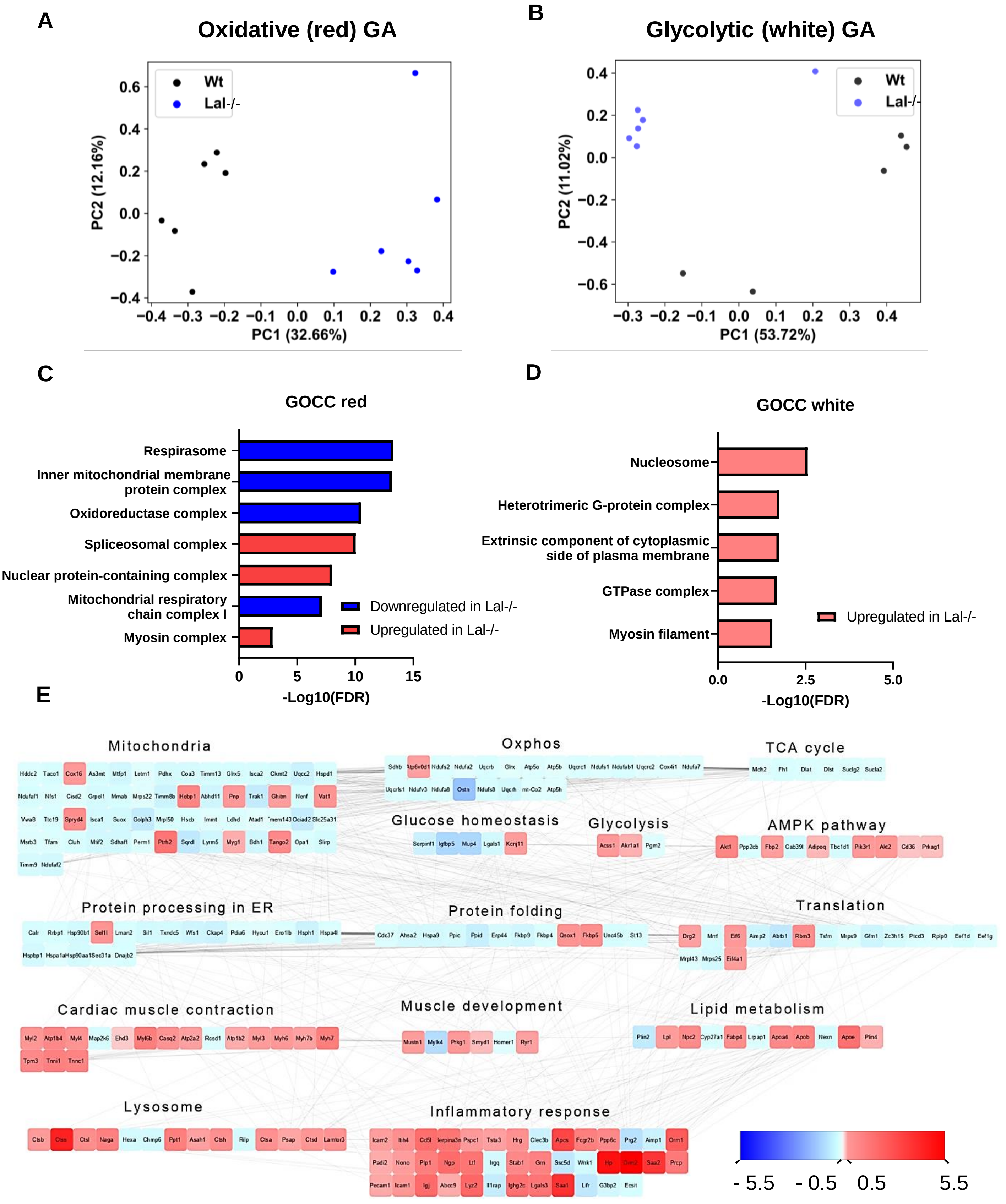

A
Oxidative (red) GA
Wt
Lal-/-
PC2 (12.16%)
PC1 (32.66%)
0.6
0.4
0.2
0.0
−0.2
−0.4
−0.4 −0.3 −0.2 −0.1 0.0 0.1 0.2 0.3 0.4
B
Glycolytic (white) GA
Wt
Lal-/-
PC2 (11.02%)
PC1 (53.72%)
0.4
0.2
0.0
−0.2
−0.4
−0.6
−0.3 −0.2 −0.1 0.0 0.1 0.2 0.3 0.4
C
GOCC red
Respirasome
Inner mitochondrial membrane protein complex
Oxidoreductase complex
Spliceosomal complex
Nuclear protein-containing complex
Mitochondrial respiratory chain complex I
Myosin complex
Downregulated in Lal-/-
Upregulated in Lal-/-
0 5 10 15
-Log10(FDR)
D
GOCC white
Nucleosome
Heterotrimeric G-protein complex
Extrinsic component of cytoplasmic side of plasma membrane
GTPase complex
Myosin filament
Upregulated in Lal-/-
0.0 2.5 5.0
-Log10(FDR)
E
Mitochondria
Oxphos
TCA cycle
Glucose homeostasis
Glycolysis
AMPK pathway
Protein processing in ER
Protein folding
Translation
Cardiac muscle contraction
Muscle development
Lipid metabolism
Lysosome
Inflammatory response
- 5.5
- 0.5
0.5
5.5

Figure S6. Top selected GOCC terms associated with proteins enriched in SM of Lal-/- mice. Principal component analysis (PCA) of the proteome with a clear separation in **(A)** oxidative or **(B)** glycolytic parts of the GA from Wt and Lal-/- mice. Top six selected gene ontology cellular component (GOCC) terms downregulated (blue) and upregulated (red) in **(C)** oxidative and **(D)** glycolytic parts of GA in young fed Wt and Lal-/- mice. **(E)** Protein–protein interaction network represented by the modulation of the significantly changed proteins in the oxidative part of GA. The interaction network was generated using Cytoscape.

Supplementary Table S1. Primer sequences used for real-time PCR

**Gene**	**Forward sequence (5’-3’)**	**Reverse sequence (5’-3’)**
Igf1	CACATCATGTCGTCTTCACACC	GGAAGCAACACTCATCCACAATG
Cd36	GCAGGTCTATCTACGCTGTG	GGTTGTCTGGATTCTGGAGG
Fatp1	CGCTTTCTGCGTATCGTCTG	GATGCACGGGATCGTGTCT
Fabp3	ACCTGGAAGCTAGTGGACAG	TGATGGTAGTAGGCTTGGTCAT
Fabp4	AAGGTGAAGAGCATCATAACCCT	TCACGCCTTTCATAACACATTCC
Cpt1b	TTCAACACTACACGCATCCC	GCCCTCATAGAGCCAGACC
Acsl1	ACCAGCCCTATGAGTGGATTT	CAAGGCTTGAACCCCTTCTG
Acox1	TCCAGACTTCCAACATGAGGA	CTGGGCGTAGGTGCCAATTA
Pgc1α	CCCTGCCATTGTTAAGACC	TGCTGCTGTTCTTGTTTTC
Pparg	GGAAGACCACTCGCATTCCTT	GTAATCAGCAACCATTGGGTCA
Murf1	GCTGGTGGAAAACATCATTGACAT	CATCGGGTGGCTGCCTTT
Atrogin1	CTTTCAACAGACTGGACTTCTCGA	CAGCTCCAACAGCCTTACTACGT
Myh1	CTCTTCCCGCTTTGGTAAGTT	CAGGAGCATTTCGATTAGATCCG
Myh2	GCACCCATCCTCATTTCGTGA	GGAATGGCACTTGCGTTTAACA
Myh3	CCAAAACCTACTGCTTTGTGGT	GGGTGGGTTCATGGCATACA
Myh4	CTTTGCTTACGTCAGTCAAGGT	AGCGCCTGTGAGCTTGTAAA
Myh7	AGACTGTCAACACTAAGAGGGT	TGCCCCAAAATGGATTCGGAT
Cyclophilin A	CCATCCAGCCATTCAGTCTT	TTCCAGGATTCATGTGCCAG
16S	CCGCAAGGGAAAGATGAAAGAC-	TCGTTTGGTTTCGGGGTTTC
Hk2	GCCAGCCTCTCCTGATTTTAGTGT	GGGAACACAAAAGACCTCTTCTGG

Supplementary Table S2. Gradient program for plasma amino acid separation by HPLC.

	Time [min]										
**Mobile Phase**	0	15	20	24	26	34	38	40	42	42,1	49
**A [%]**	86	86	70	65	53	50	30	0	0	86	86
**B [%]**	14	14	30	35	47	50	70	100	100	14	14
Flow rate: 1.1 ml / min											
